# Curcumin Derivatives as Potential Mosquito Larvicidal Agents against Two Mosquito Vectors, *Culex pipiens* and *Aedes albopictus*

**DOI:** 10.3390/ijms22168915

**Published:** 2021-08-18

**Authors:** Dimitris Matiadis, Panagiota G. V. Liggri, Eftichia Kritsi, Niki Tzioumaki, Panagiotis Zoumpoulakis, Dimitrios P. Papachristos, George Balatsos, Marina Sagnou, Antonios Michaelakis

**Affiliations:** 1 Institute of Biosciences and Applications, National Centre for Scientific Research “Demokritos”, 15310 Athens, Greece; matiadis@bio.demokritos.gr; 2 Department of Biochemistry and Biotechnology, University of Thessaly, Biopolis, 41500 Larissa, Greece; p.liggri@bpi.gr (P.G.V.L.); nikitzioumaki@gmail.com (N.T.); 3 Benaki Phytopathological Institute, Scientific Directorate of Entomology and Agricultural Zoology, 14561 Kifissia, Greece; d.papachristos@bpi.gr (D.P.P.); g.balatsos@bpi.gr (G.B.); 4 Institute of Chemical Biology, National Hellenic Research Foundation, 48 Vas. Constantinou Avenue, 11635 Athens, Greece; ekritsi@eie.gr (E.K.); pzoump@uniwa.gr (P.Z.); 5 Department of Food Science and Technology, University of West Attica, Ag. Spyridonos, 12243 Egaleo, Greece

**Keywords:** curcumin, curcuminoids, larvicidal, common house mosquito, Asian tiger mosquito, in silico descriptors

## Abstract

Vector-borne diseases have appeared or re-emerged in many Southern Europe countries making the transmission of infectious diseases by mosquitoes (vectors) one of the greatest worldwide health threats. Larvicides have been used extensively for the control of *Aedes (Stegomyia) albopictus* (Skuse, 1895) (Diptera: Culicidae) and *Culex pipiens* Linnaeus, 1758 (Diptera: Culicidae) mosquitoes in urban and semi-urban environments, causing the increasing resistance of mosquitoes to commercial insecticides. In this study, 27 curcuminoids and monocarbonyl curcumin derivatives were synthesised and evaluated as potential larvicidal agents against *Cx. pipiens* and *Ae. albopictus*. Most of the compounds were more effective against larvae of both mosquito species. Four of the tested compounds, curcumin, demethoxycurcumin, curcumin-BF_2_ complex and a monocarbonyl tetramethoxy curcumin derivative exhibited high activity against both species. In *Cx. pipiens* the recorded LC_50_ values were 6.0, 9.4, 5.0 and 32.5 ppm, respectively, whereas in *Ae. albopictus* they exhibited LC_50_ values of 9.2, 36.0, 5.5 and 23.6 ppm, respectively. No conclusive structure activity relationship was evident from the results and the variety of descriptors values generated in silico provided some insight to this end.

## 1. Introduction

Diseases caused from vector bites such as mosquitos, ticks and fleas have more than tripled in the United States from 2004–2016 [1]. Among them, mosquito-borne diseases (MBDs) present a major challenge and concern of global public health and safety. Travel and trade globalization, unplanned urbanization and environmental change have had a significant impact on disease transmission in recent years [2,3]. Regarding diseases caused by *Aedes*-borne viruses, more than one million Zika virus (ZIKV) infections and thousands of infants with birth defects were reported from 2015–2016 and similarly Dengue fever (DENV) which infects over one hundred million people in over 100 countries every year [4,5]. In 1999, the West Nile Virus (WNV) crisis across the United States with hundreds of deaths annually are some further examples of MBDs transmitted to humans usually through the bite of an infected mosquito of the genus *Culex* [6]. Finally, except human activities, the climate change has also caused mosquitoes to move globally, facilitating the transmission of MBDs [7].

*Aedes albopictus*, commonly known as the Asian tiger mosquito, is an endemic species of Africa, Asia and South America. In Europe is considered as an invasive mosquito species but it has already been established in many countries [8]. This mosquito species is anthropophilic, a day biting mosquito species, highly adaptable in the environment and can survive in both rural and urban areas. Consequently, it is a species which lives in close proximity to humans, developing preferably in urban and suburban areas where human hosts are readily available [9,10]. On the other hand, *Culex pipiens*, also known as the common house mosquito, is a widespread mosquito species which plays an important role in transmitting many human pathogens such as WNV [11,12,13] and it fits the stereotype of the “domestic” mosquito. This mosquito species thrives in highly contaminated sewers, mates in confined spaces that often enter homes and feeds easily on mammals, especially humans. Moreover, many researchers have attributed its global distribution and abundance to its ability to exploit different modes of human transport [12,13].

For many of the aforementioned MBDs the integrated vector control strategy includes the use of synthetic chemicals as a primary approach while new alternative strategies have been proposed and evaluated mainly for invasive species [14]. Despite the quick action killing of these chemicals, their repeated use has led to the development of resistance and adverse effects to non-target organisms and the environment [15]. As an alternative strategy the use of plant-derived products has been suggested as an additional group of potential larvicidal agents due to their rapid biodegradability, eco-friendliness and superior safety profile [16].

To this end, the essential oils from the Dai medicinal plant *Zingiber cassumunar* against *Ae. albopictus* exhibited interesting repellent, larvicidal and adulticidal activity. The activity observed was primarily attributed to the presence of the (−)-terpinen-4-ol of the extract [17]. Additionally, the work by Zhu et al. presented the evaluation of the larvicidal activity of four plant essential oils—cinnamon oil, lemon eucalyptus oil, sandalwood oil and turmeric oil—against 4th instars of *Ae. albopictus*, *Ae. aegypti* and *Cx. pipiens*. They also evaluated the activity of some individual oil components. Despite the promising larvicidal activities some acute toxicity was observed [18]. Based on the thorough review on natural products as leads to potential mosquitocides by Koshore et al. [19] a wide variety of naturally derived compounds have been evaluated for their larvicidal and mosquitocidal activities. In order to highlight any possible mechanism-based activity it collectively organized the compounds according to the chemical structural classes they belong including alkanes, alkenes, alkynes and simple aromatics, essential oils and fatty acids, terpenoids, steroids, alkaloids, naphthoquinones, lignans, coumarins, retinoids, flavonoids and isoflavonoids and finally, phenolic acids and curcuminoid.

Regarding curcuminoids, *Curcuma longa* is a traditional Chinese herb belonging to the Zingiberaceae family and curcumin is the active ingredient of its rhizome extract. Many literature reports have previously presented and discussed the larvicidal activities against *Ae. aegypti*, *Ae. albopictus*, *Anopheles gambiae* and others of the essential oil, various extracts and some of their constituents of this plant [18,20,21,22,23,24,25]. More specifically, our group has previously evaluated the larvicidal activity of the 3 curcuminoids, curcumin, demethoxycurcumin and bis-demethoxycurcumin, isolated from the natural mixture and three synthetic derivatives against *Cx. pipiens*. Curcumin and di-O-demethylcurcumin exhibited significant potency with LC_50_ value of 19.07 and 12.42 mg/L, respectively [26].

**Figure 1:**
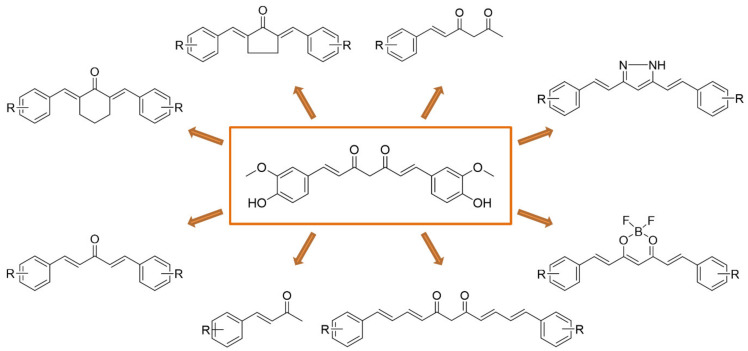
Generalised structures of the synthetic curcumin derivatives studied herein.

Aiming to improve the larvicidal activity against *Cx. pipiens* as well as evaluate their potential activity against an invasive mosquito species (*Ae. albopictus*), we would like to report herein the synthesis of a small library of curcumin derivatives. A variety of structural modifications of the original curcumin skeleton were made, including the removal of one of the two carbonyl moieties, resulting in monocarbonyl derivatives, the removal of one half of the molecule, polyhydroxylated aromatic substitution, following our previous results, the addition of double bonds and the boron-coordination of the diketone moiety with BF_2_. Figure 1 summarises the general structural modification of the curcumin derivatives studied herein as potential larvicidals against *Ae. albopictus* and *Cx. pipiens.*

## 2. Results and Discussion

### 2.1. Synthesis

**Scheme 1:**
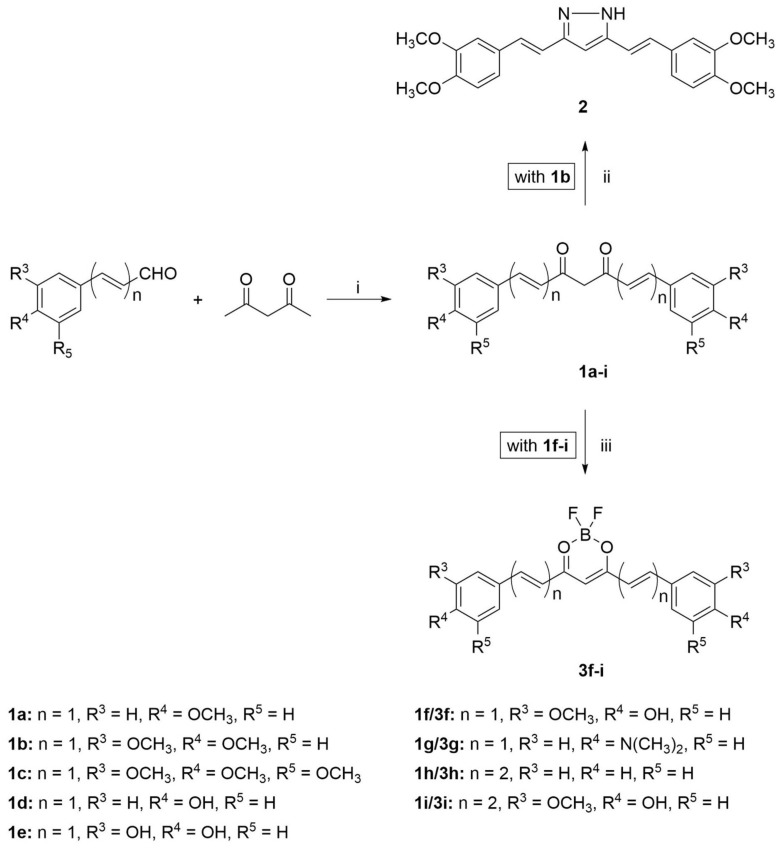
Synthesis of symmetric dicarbonyl analogues of curcumin **1a–i**, pyrazole derivative 2 and boron difluoride complexes **3f–i**. Reagents and conditions: (**i**) B_2_O_3_, B[(OBu)_3_], 90 °C, 30 min; then, addition of nBuNH_2_, 70–100 °C, 24 h; (**ii**) NH_2_NH_2_·H_2_O, CH_3_COOH, EtOH, reflux, 24 h; (**iii**) BF_3_·OEt_2_, toluene, 65 °C, 5 h.

The general synthetic routes to symmetric dicarbonyl analogues of curcumin **1a–i**, along with the rigid cyclized pyrazole **2** and BF_2_ analogues **3f–i** are depicted in Scheme 1. Compounds **1a–i** were prepared according to well-established curcumin synthetic protocols with slight modifications. Boron trioxide (B_2_O_3_) was added in a mixture of tributyl borate B[(OBu)_3_], acetylacetonate and the appropriate substituted benzaldehyde to form a complex with the acetylacetone, thus ensuring that the aldol condensation occurs only at the terminal methyl groups. The reactions took place in the absence of any organic solvent and after the addition of *n*-butylamine (nBuNH_2_), the boron complexes of the final products were hydrolyzed by aqueous hydrochloric acid.

Pyrazole **2** was prepared from compound **1b** using threefold excess of hydrazine hydrate in a 1:1 acetic acid/ethanol mixture as solvent. The product was obtained as a precipitated solid in satisfactory yield and high purity without any additional purification steps.

Difluoroboron curcumin derivatives **3f–i** were prepared by the addition of boron trifluoride diethyletherate to the corresponding curcumins **1f–i**. The formation of the complexes was evidenced by the higher frequency shifts of the *β*-diketone unit active methylene proton (0.33–0.50 ppm).

**Scheme 2:**
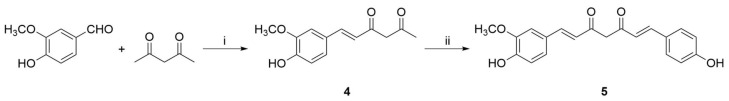
Synthesis of monoarylidene dicarbonyl derivative **4** and curcumin II (demethoxycurcumin) **5**. Reagents and conditions. (**i**) (excess of acetylacetone), vanillin, B_2_O_3_, B[(OBu)_3_], EtOAc, 80 °C, 30 min; then, addition of *n*BuNH_2_, room temperature, 24 h; (**ii**) 4-hydroxybenzaldehyde, B_2_O_3_, B[(OBu)_3_], 50 °C, 30 min; then, addition of *n*BuNH_2_, 50 °C, 24 h.

Non-symmetric curcuminoid **5** (known as curcumin III) was prepared from condensation of the monoarylidene curcumin analogue **4** with 4-hydroxybenzaldehyde (Scheme 2). **4** was synthesized using an excess of acetylacetone in ethyl acetate in order to avoid significant formation of the symmetric diarylidene derivative (**1f**).

**Scheme 3:**
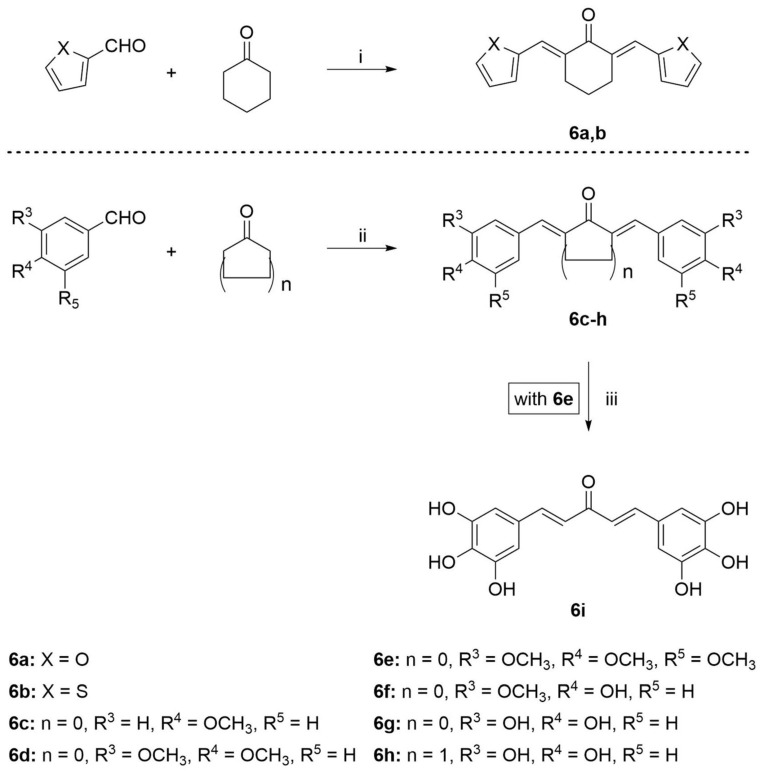
Synthesis of monocarbonyl analogues of curcumin **6a–h**. Reagents and conditions: (**i**) cyclohexanone, KOH powder, EtOH, room temperature., 2 h; (**ii**) acetone, NaOH/EtOH 10%, room temperature, 2 h (**6c–e**) or acetone (or cyclopentanone), c. HCl, EtOH, room temperature, 24 h (**6f–h**); (**iii**) BBr_3_, DCM (dry), −20 °C to 0 °C to room temperature, 3 h.

Compounds **6a–h** were obtained by Claisen–Schmidt condensation of the appropriate aromatic aldehydes and cyclohexanone or acetone using a ratio of 1:2 of ketone to aldehyde under alkaline or acidic conditions (Scheme 3). Thiophenyl and furanyl derivatives **6a,b** were obtained in best purity and yields using solid potassium hydroxide, while for methoxy-substituted phenyl derivatives **6c–e**, a dispersion of sodium hydroxide in ethanol was added instead. Dihydroxybenzaldehyde and vanillin derived analogues **6f–h** were prepared under acidic conditions using concentrated hydrochloric acid. Finally, polyhydroxylated compound **6i** was obtained by demethylation of **6e** with boron tribromide in anhydrous dichloromethane (DCM).

**Scheme 4:**
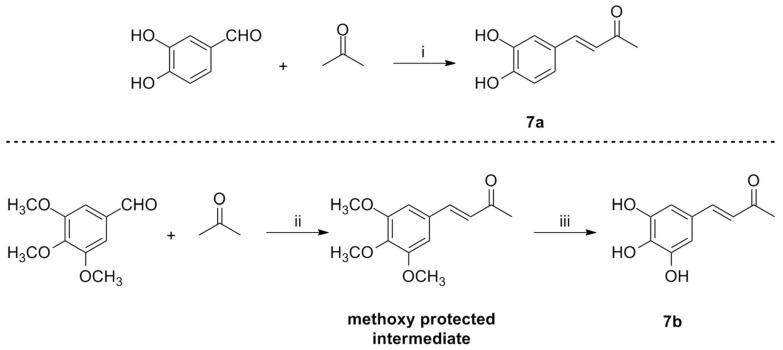
Synthesis of monoarylidene dicarbonyl derivatives **7a** and **7b**. Reagents and conditions: (**i**) c. HCl, EtOH, r.t., 24 h; (**ii**) aq. NaOH 1M, r.t., 24 h; (**iii**) BBr_3_, DCM (dry), −20 °C to 0 °C to r.t., 3 h.

Similarly, to **6g** and **6i**, half curcuminoids (monoarylidene) **7a** and **7b** were prepared by Claisen-Schmidt condensation between 3,4-dihydroxybenzaldehyde and acetone (**7a**) and by demethylating the intermediate trimethoxy half curcuminoid following the boron tribromide protocol (**7b**) (Scheme 4). In both cases, excess of acetone for the Claisen-Schmidt reaction was used to minimize the production of bisarylidene molecules.

The purity and structure of all synthesized compounds were determined by ^1^H NMR spectroscopy and elemental analyses. Novel derivative **3i** was further characterized by ^13^C and 2D NMR experiments.

### 2.2. Larvicidal Activity

**Figure 2:**
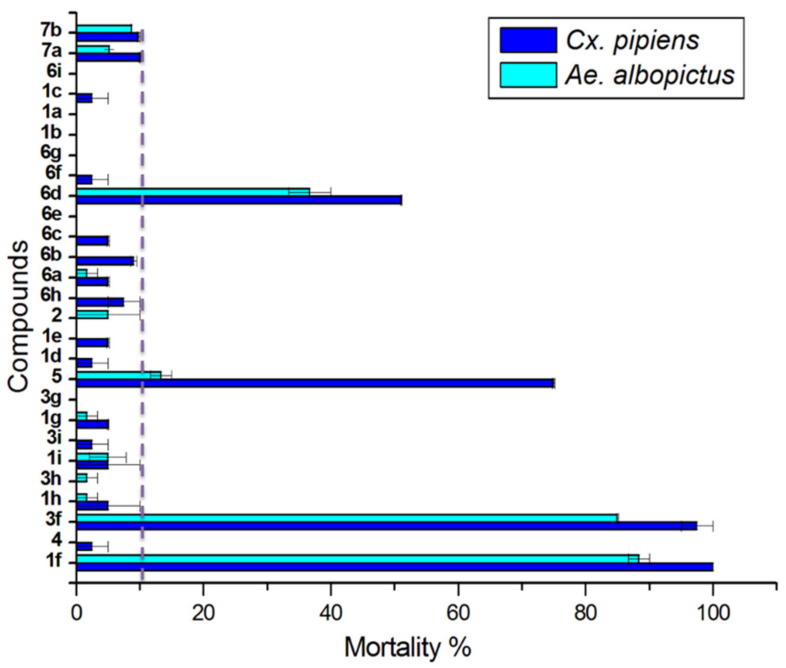
Larvae mortality against third- to fourth-instar larvae of *Aedes albopictus* and *Culex pipiens* biotype *molestus* was recorded at 20 ppm after 24 h. The dashed line shows the 10% mortality level. No mortality was observed in the control experiments. Error bars = standard error of the mortality mean value.

Initially, all compounds were screened at a concentration of 20 ppm against *Ae. albopictus* and *Cx. pipiens* for 24 h (Figure 2). No mortality was observed in the control experiments of tap water and 2% DMSO in water. Among the tested compounds, in both species, the same 4 compounds (**1f**, **3f**, **5** and **6d**) exhibited mortality ≥ 10%, regardless of the observed difference in the overall mortality effect. For the rest of the tested compounds **11** and **6** caused <10% mortality against the larvae of *Cx. pipiens* and *Ae. albopictus*, respectively, while the other derivatives were completely inactive against larvae of both species. The results were not altered at all after a 48 h incubation time with the only exception of **3f** which exhibited a very slight increase in activity.

Consequently, the 4 most active compounds revealed from the 20 ppm screening experiment, namely **1f**, **3f, 5** and **6d**, were further evaluated for larvicidal activity using various concentrations for 24 h incubation. In that way, a dose-response bioassay was able to be drawn enabling the estimation of their respective LC_50_ and LC_90_ values (Table 1).

In the case of larvicidal activity against *Cx. pipiens* compound **3f,** the BF_2_-curcumin complex, exhibited the highest activity with an LC_50_ value of 5 ppm whereas curcumin itself (**1f**) was almost equipotent with LC_50_ value of 6 (5.4–6.6) ppm. The activity of **3f** was also very slightly increased upon 48 h treatment showing an LC_50_ value of 4.2 against *Cx. pipiens.* The second curcuminoid, demethoxycurcumin, **5,** was also highly active with an LC_50_ value of 9.4 (8.2–10.6) ppm while the monocarbonyl curcumin analogue **6d** was the least active of all. Similarly, when *Ae. albopictus* larvae were incubated with **3f** the highest activity was observed followed by that of **1f**, exhibiting LC_50_ values of 5.5 (4.6–6.3) ppm and 9.2 (8.5–9.9) ppm, respectively. As before the LC_50_ value of **3f** was slightly smaller at the range of 4.8 ppm. The other two compounds, **5** and **6d**, were less active against *Ae. albopictus* larvae compared to their effect against *Cx. pipiens*, with LC_50_ values of 36 (32.6–39.5) ppm and 23.6 (21.3–26.2) ppm, respectively. Moreover, in both cases LC_90_ values follow the same order of activity.

### 2.3. Calculations of Molecular Descriptors

The larvicidal activity results against *Ae. albopictus* and *Cx. pipiens* clearly indicate that curcumin (**1f**) and their synthesized derivatives **3f**, **5** and **6d** possessed a remarkable activity, compared to their structural similar compounds. Based on the results obtained a better understanding of their structure-activity relationships was attempted by generating a plethora of descriptors the values of which are presented in Table 2.

There is no clear rationalization within the ALogP, whereas the active molecules seem to have a number of 5 or 6 hydrogen bond acceptors. Interestingly, the molar refractivity (MR) values of all active compounds fall within the range between 96.77 and 107.61 in addition to their very narrow range of molecular polarizability (Polar) which characterizes them ranging between 43.0 and 45.74. The almost equipotent compounds **1f** and **3f** seem to present a great difference in their lipophilicity values, share the same hydrogen bond donor and acceptor values and they have very close molar polarisability results.

The analysis of the calculated descriptors values revealed that the replacement of the dicarbonyl group of **1f** by cyclized pyrazole ring (compound **2**) has a negative contribution to the larvicidal activity. Particularly, the lipophilicity value (ALogP) of derivative **2** is increased (ALogP = 3.85) compared to the lipophilicity value of curcumin (**1f**) (ALogP = 3.01) and the opposite phenomenon is regarded for the electrotopological state index (E-state) value, which describes the influential molecular fragments. Especially, the E-state values for compounds **1f** and **2** are 71.84 and 63.84, respectively.

In the case of compound **1g** the methoxy (-OMe) and the hydroxyl (-OH) groups of curcumin (**1f**) have been replaced by hydrogen (-H) and amine (-NR_2_) groups, respectively. The comparison of the calculated descriptors’ values between the active compound **1f** and the inactive compound **1g** indicated greater values for the lipophilicity (ALogP = 3.90) and a reduction of hydrogen bond donors and acceptors. Furthermore, the Polar Surface Area (PSA) value of compound **1g**, which describes the van der Waals surface area of polar nitrogen and oxygen atoms, is significantly reduced (PSA = 40.62) compared to **1f** (PSA = 93.06). Comparison between curcumin (**1f**) and its symmetric dicarbonyl analogue **1i** displayed higher values for lipophilicity (ALogP = 3.94), Molar Refractivity (MR = 123.87) and Polarizability (Polar = 53.43) for **1i**.

In continuation, the comparison of the physicochemical profile of the most active difluorocurcumin derivative **3f** and its structural similar inactive compound **3g** showed that the replacement of methoxy (-OMe) and hydroxyl (-OH) groups causes an increase in lipophilicity (ALogP), Molar Refractivity (MR) and Polarizability values and a decrease in Polar Surface Area (PSA) and Electrotopological state index (E-state) values. In the described case, the greatest difference is identified in Polar Surface Area values, which are 85.22 and 32.78 for compounds **3f** and **3g**, respectively. Moreover, similar physicochemical pattern is followed for compound **3i,** in which the aliphatic chain contains one additional carbon double bonds at each side of the diketone moiety.

From compounds bearing mono-carbonyl substitution, compound **6d** presents larvicidal activity against *Ae. albopictus* and *Cx. pipiens*. Physicochemical profile comparison of derivative **6d** with its structurally similar compounds **6c**, **1b**, **6e** and **6f** indicates a notable difference in Polar Surface Area values. Indicatively, for **6d** the value is 53.99 while for compound **6c**, which bears only one methoxy group is 35.53. In addition, for compounds **1b**, **6e** and **6f** an increase is regarded.

There has been, for years, a significant amount of research efforts trying to identify the most potent larvicidal extract of various sources of Curcuma spices but only very few of them isolated, identified and evaluated the activity of individual compounds. The crude methanolic extract of *C. longa* gave an LC_50_ value against the 4th-instar larvae of *Cx. pipiens pallens* of 355.06 ppm [25]. This study revealed the larvicidal activity of ar-turmerone and 8-hydroxyl-ar-turmerone. The two compounds exhibited larvicidal activities against the 4th-instar larvae of *Cx. pipiens pallens* after 24 h of treatment with LC_50_ values of 138.86 and 257.68 ppm, respectively. The petroleum ether extract of *C. aromatic* eliminated *Cx. quinquefasciatus* at an LC_50_ value of 11.42 ppm [23]. Further investigation of the extract lead to the isolation of two larvicidal compounds namely 9-oxoneoprocurcumenol and neoprocurcumenol. The former exerted significant toxicity (*p* < 0.01) on mosquito larvae with LC_50_ value of 5.81 ppm compared to the latter with 13.69 ppm. However, bearing in mind that active phytochemicals can be influenced by multiple factors, such as the type of solvent used for the extraction and the extraction process, the plant genus, the conditions under which the plants were harvested and the target mosquito species used for the tests [27] it is important to identify more synthetic derivatives of such active natural products.

In the present study, 27 synthetic analogues and derivatives of curcumin were evaluated against *Cx. pipiens* and *Ae. albopictus* larvae and only 4 of them exerted significant larvicidal activity one of them being curcumin itself, **1f**. Its activity against *Cx. pipiens* larvae was found to have a small deviation from our previous results [26], showing an increased activity with an LC_50_ value of 6 ppm compared to 19 ppm. Additionally, demethoxycurcumin, **5**, had been previously found inactive against *Cx. pipiens* whereas an exciting LC_50_ value of 9.4 ppm was exerted in this study further to the equally significant LC_50_ value of 36.0 ppm against *Ae. albopictus*. The small discrepancies from our previous results may be related to the relative differences in the developmental stage of the mosquitoes in the two studies. It has been reported that certain biochemical changes to some target molecules from the first to the fourth instar, such as sterol carrier protein-2, acetylcholinesterase, detoxification and resistance mechanisms and others, may affect treatment results [28]. The same was observed for the methanol crude extract of *Artemisia nilagirica* (Clarke) with reported LC_50_ values of 272.50, 311.40, 361.51 and 442.51 ppm, respectively, against the first to fourth instar larvae of *An. stephensi* and 300.84, 338.79, 394.69 and 470.74 ppm, respectively, against the first to fourth instar larvae of *Ae. aegypti*. [29] Moreover, our overall observation for the lower activity exhibited by all tested compounds against *Ae. albopictus* compared with *Cx. pipiens* may also be related to analogous interspecies variations. Similar results have been reported for the natural compound palmitic acid, extracted from *Millettia pinnata* (L.) seeds, which showed LC_50_ values of 34.50, 42.96 and 85.61 ppm against the third instar larvae of *Cx. pipiens pallens*, *Ae. aegypti* and *Ae. albopictus*, respectively [30].

To our surprise any change to the curcumin skeleton eliminated larvicidal effect against both species. This was found to be the case regardless if the change was at the substitution pattern or functional group substituent or the presence of additional double bond. The only structural transformation which benefited the activity of the molecule was the formation of the BF_2_ complex, **3f**, which increased larval mortality even more compared to its mother compound, **1f**. It is noteworthy that none of the inactive diketonic curcuminoids exerted higher activity when converted to its corresponding BF_2_ complex which implies that the curcumin part dictates the degree of activity. In the case of **3f**, therefore, the anticipated increased chemical, photo- and pH stability and improved solubility compared to **1f** may certainly account for the increased larvicidal effect observed, especially in the case of *Ae. albopictus*, as well as the slightly higher activity exerted after the 48-h exposure.

Based on our previous work in which the tetrahydroxycurcumin, **1e** exerted significant larvicidal effect it was part of our initial hypothesis to investigate the activity of some polyhydroxylated monocarbonyl analogues of curcumin of the type of **6a,b** and **7a,b** in comparison to **1d** and **1e**. [26]. The lack of chemical stability, the rapid degradation and poor bioavailability of curcuminoids has been strongly related to the β-diketone moiety [31,32]. In recent years, the solution to the aforementioned limitations has been intensely explored in the synthesis and application of monocarbonyl derivatives which have shown indeed improved biological and pharmacological profile [33,34]. In our case, however, neither the polyhydroxylated monocarbonyl derivatives showed any activity at all nor the diketone counterparts. On the contrary, significant larvicidal activity was recorded against both mosquito species by the tetramethoxy monocarbonyl curcumin derivative, **6d**. To the best of our knowledge, only the work by Anstrom [35] has searched into similar monocarbonyl curcumin derivatives as potential mosquitocidal agents and more specifically it made an effort to relate the inhibitory potential against sterol carrier protein-2, although a clear correlation was not found. This type of monocarbonyl derivatives, due to the ease of preparation and low production cost, should be, therefore, investigated further aiming to elucidate some more mechanistic information on the mode of their larvicidal action. The structurally related chalcones have also been investigated as mosquito larvicides and the compound (E)-3-(4-bromophenyl)-1-(furan-2-yl) prop- 2-en-1-one exhibited an LC_50_ value of 6.66 mg/L at 24 h against *Ae. aegypti* larvae [36]. Additionally, 28 compounds, chalcones and some derived products, were synthesized and tested for mosquito larvicidal activity against the third instar larvae of *Cx. quinquefasciatus.* Four of them exhibited outstanding activity of 5–55 μM and some structure-activity relationship was derived [37]. Finally, the increased larvicidal activities of some chalcones against *Ae. albopictus* with LC_90_ values of 5 ppm after 72 h of exposure have been related to their high activity as juvenile hormone antagonist (JHAN) [38]. From a mechanistic point of view, acetylcholinesterase (AChE) is considered as the most interesting molecular target because it is a critical nervous system enzyme responsible for synaptic transmission and is the target site for organophosphoate and carbamate insecticides [31,39]. Additionally, interference with the octopaminergic system has been related to the insecticidal activity of natural products [30]. Finally, the larvicidal mechanism of ar-turmerone has been attributed to stomach poisoning and the active sites might be the muscle and digestive tissues [40]. Further investigation is necessary and currently underway to provide some evidence on the mode of action of our compounds.

In conclusion, in our hands 4 out of 27 curcuminoids exhibited highly promising larvicidal activity against *Ae. albopictus* and *Cx. pipiens.* Further chemical derivatisation of curcumin skeleton is underway to optimize physicochemical properties and larvicidal activity in laboratory and field conditions as well as to provide further understanding of their mechanism of action.

## 3. Methods and Materials

### 3.1. General

All reagents were purchased from Sigma-Aldrich (St. Louis, MO, USA), Alfa Aesar (Lancaster, UK) and TCI (Tokyo, Japan) and used without further purification. NMR spectra were recorded with a Bruker Avance 500 MHz spectrometer (Bruker, Rheinstetten, Germany) operating at 500 MHz (^1^H) and 125 MHz (^13^C). Chemical shifts are reported in ppm relative to DMSO-*d*_6_ (^1^H: δ = 2.50 ppm, ^13^C: δ = 39.52 ± 0.06 ppm). The following are included in the Supplementary Information file; Figures S1–S13, S13–S19: ^1^H NMR spectra of known compounds, Figures S14–S18: 1D and 2D NMR spectra of novel compound **3i**. Elemental analyses were performed using a PerkinElmer 2400 CHNS Organic Elemental Analyzer 100 V (PerkinElmer Inc., Boston, MA, USA).

### 3.2. Synthesis

#### 3.2.1. Synthesis of Compounds **1a–i**

Boron trioxide (0.35 g, 5.0 mmol), tributyl borate (10.8 mL, 40 mmol), acetylacetonate (1.03 mL, 10.0 mmol) and the appropriate aromatic aldehyde (20 mmol) were added in a 50 mL round-bottom flask. The mixture was stirred at 90 °C for 30 min. *n*-Butylamine (0.4 mL, 4.0 mmol) was added dropwise over 30 min at 70 °C. After the addition, the mixture was stirred at 100 °C for 90 min and then at 70 °C for 16 h. Hydrochloric acid (30 mL, 1 M) was added and the mixture was stirred at 60 °C for 2 h. The mixture was extracted with ethyl acetate (3 × 30 mL). The combined organic extracts were washed with brine, dried with Na_2_SO_4_ and concentrated under vacuum. The crude products were purified by MeOH (**1a–c,g**), EtOH (**1d,f,h**) or flash chromatography (CHCl_3_:MeOH = 100/0 to 90/10).

(1*E*,6*E*)-1,7-bis (4-methoxyphenyl) hepta-1,6-diene-3,5-dione **1a** [41]
Orange solid, Yield: 1.46 g (44%); ^1^H NMR (500 MHz, DMSO-*d*_6_): δ 3.81 (s, 6H), 6.09 (s, 1H), 6.80 (d, 2H, *J* = 15.9 Hz), 7.01 (d, 4H, *J* = 8.6 Hz), 7.60 (d, 2H, *J* = 15.9 Hz), 7.69 (d, 4H, *J* = 8.5 Hz); Anal. Calcd for C_21_H_20_O_4_: C, 74.98, H, 5.99. Found C, 74.93, H, 6.05.
(1*E*,6*E*)-1,7-bis (3,4-dimethoxyphenyl) hepta-1,6-diene-3,5-dione **1b** [26]
Orange powder, Yield: 2.18 g (55%); ^1^H NMR (500 MHz, DMSO-*d*_6_): δ 3.81 (s, 6H), 3.83 (s, 6H), 6.11 (s, 1H), 6.84 (d, 2H, *J* = 15.9 Hz), 7.02 (d, 2H, *J* = 8.3 Hz), 7.27 (d, 2H, *J* = 8.3 Hz), 7.35 (s, 2H), 7.59 (d, 2H, *J* = 15.9 Hz); Anal. Calcd for C_23_H_24_O_6_: C, 69.68, H, 6.10. Found C, 69.62, H, 6.14.
(1*E*,6*E*)-1,7-bis(3,4,5-trimethoxyphenyl) hepta-1,6-diene-3,5-dione **1c** [42]
Orange solid, Yield: 1.87 mg (40%); ^1^H NMR (500 MHz, DMSO-*d*_6_): δ 3.71 (s, 6H), 3.84 (s, 12H), 6.18 (s, 1H), 6.94 (d, *J* = 16.0 Hz, 2H), 7.01 (s, 4H), 7.59 (d, *J* = 16.0 Hz, 2H); Anal. Calcd for C_25_H_28_O_8_: C, 65.78, H, 6.18. Found C, 65.84, H, 6.24.
(1*E*,6*E*)-1,7-bis (4-hydroxyphenyl) hepta-1,6-diene-3,5-dione **1d** [43]
Orange solid, Yield: 1.61 g (52%); ^1^H NMR (500 MHz, DMSO-*d*_6_): δ 6.04 (s, 1H), 6.70 (d, *J* = 15.8 Hz, 2H), 6.82 (d, *J* = 8.3 Hz, 4H), 7.55–7.57 (m, 4H), 10.05 (br, 2H); Anal. Calcd for C_19_H_16_O_4_: C, 74.01, H, 5.23. Found C, 73.94, H, 5.26.
(1*E*,6*E*)-1,7-bis (3,4-dihydroxyphenyl) hepta-1,6-diene-3,5-dione **1e** [26]
Orange to red solid, Yield: 1.60 g (47%); ^1^H NMR (500 MHz, DMSO-*d*_6_): δ 6.04 (1H), 6.56 (*J* = 15.6 Hz, 2H), 6.77 (*J* = 8.3 Hz, 2H), 7.00 (*J* = 1.7 Hz, 2H), 7.07 (*J* = 8.3, 1.7 Hz, 2H), 7.46 (*J* = 15.6 Hz, 2H), 10.08 (br, 4H); Anal. Calcd for C_19_H_16_O_6_: C, 67.06, H, 4.74. Found C, 67.01, H, 4.71.
(1*E*,6*E*)-1,7-bis (4-hydroxy-3-methoxyphenyl) hepta-1,6-diene-3,5-dione **1f** [43]
Orange solid, Yield: 2.34 g (64%); ^1^H NMR (500 MHz, DMSO-*d*_6_): δ 3.83 (s, 6H), 6.06 (s, 2H), 6.76 (d, *J* = 15.8 Hz, 2H), 6.82 (d, *J* = 8.0 Hz, 2H), 7.15 (d, *J* = 8.0 Hz, 2H), 7.32 (s, 2H), 7.55 (d, *J* = 15.8 Hz, 2H), 9.65 (s, 4H); Anal. Calcd for C_21_H_20_O_6_: C, 68.47, H, 5.47. Found C, 68.41, H, 5.51.
(1*E*,6*E*)-1,7-bis (4-(dimethylamino)phenyl) hepta-1,6-diene-3,5-dione **1g** [43]
Dark red solid, Yield: 2.42g (67%); ^1^H NMR (500 MHz, DMSO-*d*_6_): 5.96 (s, 1H), 6.60 (d, *J* = 15.8 Hz, 2H), 6.73 (d, *J* = 8.8 Hz, 4H), 7.49–7.54 (m, 6H); Anal. Calcd for C_23_H_26_N_2_O_2_: C, 76.21, H, 7.23, N, 7.73. Found C, 76.23, H, 7.16, N, 7.69.
(1*E*,3*E*,8*E*,10*E*)-1,11-diphenylundeca-1,3,8,10-tetraene-5,7-dione **1h** [43]
Yellow to orange solid, Yield: 2.05 g (63%); ^1^H NMR (500 MHz, DMSO-*d*_6_): δ 6.09 (s, 2H), 6.38 (d, *J* = 15.1 Hz, 2H), 7.23–7.07 (m, 4H), 7.50–7.30 (m, 8H), 7.59 (d, *J* = 7.4 Hz, 4H); Anal. Calcd for C_23_H_20_O_2_: C, 84.12, H, 6.14. Found C, 84.16, H, 6.09.
(1*E*,3*E*,8*E*,10*E*)-1,11-bis(4-hydroxy-3-methoxyphenyl)undeca-1,3,8,10-tetraene-5,7-dione **1i** [44]
Deep red solid, Yield: 1.75 mg (42%); ^1^H NMR (500 MHz, DMSO-*d*_6_): δ 3.82 (s, 6H), 6.00 (s, 2H), 6.26 (d, *J* = 15.1 Hz, 2H), 6.78 (d, *J* = 7.8 Hz, 2H), 6.98–7.01 (m, 6H), 7.19 (s, 2H), 7.38–7.42 (m, 2H), 9.47 (s, 2H); Anal. Calcd for C_25_H_24_O_6_: C, 71.42, H, 5.75. Found C, 71.39, H, 5.80.

#### 3.2.2. Synthesis of Compound **2**

To a solution of compound **1b** (0.99 g, 2.5 mmol) in acetic acid (10 mL) and ethanol (12 mL), hydrazine hydrate (0.24 g, 7.5 mmol) was added. The solution was stirred under reflux for 24 h. Ethanol was evaporated under vacuum and the resulting solution was added to a mixture of ice and water (50 mL). The precipitate was filtered, washed with water (3 × 5 mL) and dried under vacuum and P_2_O_5_.

3,5-bis ((E)-3,4-dimethoxystyryl)-1H-pyrazole **2** [45]
White to light pink solid, Yield: 905 mg (92%); ^1^H NMR (500 MHz, DMSO-*d*_6_): δ 3.77 (s, 3H), 3.82 (s, 3H), 6.65 (s, 1H), 6.91–6.99 (m, 3H), 7.03–7.09 (m, 5H), 7.15–7.21 (m, 2H), 12.88 (s, 1H); Anal. Calcd for C_23_H_24_N_2_O_4_: C, 70.39, H, 6.16, N, 7.14. Found C, 70.43, H, 6.10, N, 7.17.

#### 3.2.3. Synthesis of Compounds **3f–i**

To a solution of compound **1f–i** (0.6 mmol) in toluene (5 mL), boron trifluoride diethyl etherate (0.11 mL, 0.9 mmol) was added. The solution was stirred at 65 °C for 5 h. The formed precipitate was filtered, washed with toluene (2 × 5 mL) and *n*-hexane (2 × 5 mL) and dried under vacuum and P_2_O_5_.

(1*E*,6*E*)-1,7-bis (4-hydroxy-3-methoxyphenyl) hepta-1,6-diene-3,5-dione-BF_2_ complex **3f** [46]
Dark red solid, Yield: 117 mg (47%); ^1^H NMR (500 MHz, DMSO-*d*_6_): δ 3.85 (s, 6H), 6.45 (s, 1H), 6.88 (d, *J* = 8.1 Hz, 2H), 7.02 (d, *J* = 15.6 Hz, 2H), 7.34 (d, *J* = 8.1 Hz 2H), 7.47 (s, 2H), 7.92 (d, *J* = 15.6 Hz, 2H), 10.09 (s, 2 H); Anal. Calcd for C_21_H_19_BF_2_O_6_: C, 60.61, H, 4.60. Found C, 60.63, H, 4.55.
(1*E*,6*E*)-1,7-bis (4-(dimethylamino)phenyl) hepta-1,6-diene-3,5-dione-BF_2_ complex **3g** [46] Black solid, Yield: 88 mg (36%); ^1^H NMR (500 MHz, DMSO-*d*_6_): δ 3.07 (s, 12H), 6.29 (s, 2H), 6.75 (d, *J* = 15.4 Hz, 2H), 6.79 (d, *J* = 8.9 Hz, 4H), 7.70 (d, *J* = 8.0 Hz, 4H), 8.01 (d, *J* = 15.4 Hz, 2H); Anal. Calcd for C_23_H_25_BF_2_N_2_O_2_: C, 67.33, H, 6.14, N, 6.83. Found C, 67.36, H, 6.10, N, 6.87.
(1*E*,3*E*,8*E*,10*E*)-1,11-diphenylundeca-1,3,8,10-tetraene-5,7-dione-BF_2_ complex **3h** [46]
Black solid, Yield: 96 mg (42%); ^1^H NMR (500 MHz, DMSO-*d*_6_): δ 6.59 (d, *J* = 15.0 Hz, 2H), 6.62 (s, 1H), 7.30–7.46 (m, 11H), 8.21 (2 × d, *J* = 7.1 Hz, 4H), 7.84 (dd, *J* = 10.6 Hz, J = 15.0 Hz, 2H); Anal. Calcd for C_23_H_19_BF_2_O_2_: C, 73.43, H, 5.09. Found C, 73.39, H, 5.12.
(1*E*,3*E*,8*E*,10*E*)-1,11-bis (4-hydroxy-3-methoxyphenyl) undeca-1,3,8,10-tetraene-5,7-dione-BF_2_ complex **3i**
Black solid, Yield: 128 mg (45%); ^1^H NMR (500 MHz, DMSO-*d*_6_): δ 3.84 (s, 6H), 6.43 (d, *J* = 14.8 Hz, 2H), 6.49 (s, 1H), 8.10 (d, *J* = 8.1 Hz, 2H), 6.97 (s, 2H), 7.07–7.30 (m, 12H), 7.77 (dd, *J* = 11.2 Hz, *J* = 15.8 Hz, 2H); ^13^C NMR (125 MHz, DMSO-*d*_6_): δ 55.7, 110.8, 115.8, 122.3, 123.2, 124.5, 127.5, 127.6, 127.5, 127.6, 146.1, 147.9, 148.0, 149.5, 178.0. Anal. Calcd for C_25_H_23_BF_2_O_6_: C, 64.13, H, 4.95. Found C, 64.15, H, 4.90.

#### 3.2.4. Synthesis of Compound **4**

Boron trioxide (0.69 g, 10.0 mmol) and acetylacetone (4.12 mL, 40 mmol) were dissolved in EtOAc (30 mL) and stirred at 50 °C for 30 min. To this mixture, tributyl borate (5.4 mL, 20 mmol) and vanillin (1.52 g, 10 mmol) were added. After stirring for 15 min, n-butylamine (0.73 mL, 10.0 mmol) solution in EtOAc (5 mL) was added dropwise. The mixture was stirred at 50 °C for 24 h. Hydrochloric acid (30 mL, 1 M) was added and the mixture was stirred at 50 °C for 3 h. The mixture was extracted with ethyl acetate (3 × 30 mL). The combined organic extracts were washed with brine, dried with Na_2_SO_4_ and concentrated under vacuum. The crude product was purified by flash chromatography (CHCl_3_) and then by recrystallization (CHCl_3_ and n-hexane).

(*E*)-6-(4-hydroxy-3-methoxyphenyl) hex-5-ene-2,4-dione **4** [47]
Pale yellow solid, Yield: 730 mg (35%); ^1^H NMR (500 MHz, DMSO-*d*_6_): δ 2.12 (s, 3H), 3.82 (s, 3H), 5.84 (s, 1H), 6.65 (d, *J* = 15.8 Hz, 1H), 6.80 (d, *J* = 8.1 Hz, 1H), 7.11 (d, *J* = 8.1 Hz, 1H), 7.29 (s, 1H), 7.49 (d, *J* = 15.9 Hz, 1H), 9.61 (s, 1H); Anal. Calcd for C_13_H_14_O_4_: C, 66.66, H, 6.02. Found C, 66.71, H, 5.97.

#### 3.2.5. Synthesis of Compound **5**

Boron trioxide (0.06 g, 0.085 mmol) and compound **4** (0.4 mL, 1.71 mmol) were dissolved in EtOAc (15 mL) and stirred at 50 °C for 1 h. To this mixture, tributyl borate (0.46 mL, 1.71 mmol) and 4-hydroxybenzaldehyde (0.21 g, 1.71 mmol) were added. After stirring for 1 h, n-butylamine (0.169 mL, 1.71 mmol) solution in EtOAc (5 mL) was added dropwise. The mixture was stirred at 50 °C for 24 h. Hydrochloric acid (30 mL, 1 M) was added and the mixture was stirred at 60 °C for 2 h. The mixture was extracted with ethyl acetate (3 × 30 mL). The combined organic extracts were washed with brine, dried with Na_2_SO_4_ and concentrated under vacuum. The crude product was purified by recrystallization from EtOAc and n-hexane and then by flash chromatography (MeOH/CHCl_3_ = 5/95).

(1*E*,6*E*)-1-(4-hydroxy-3-methoxyphenyl)-7-(4-hydroxyphenyl) hepta-1,6-diene-3,5-dione **5** [26]
Orange solid, Yield: 245 mg (42%); ^1^H NMR (500 MHz, DMSO-*d*_6_): δ 3.83 (s, 3H), 6.04 (s, 1H), 6.70 (d, *J* = 15.8 Hz, 1H), 6.75 (d, *J* = 15.8 Hz, 1H), 6.82 (m, 3H), 7.14 (d, *J* = 7.8 Hz, 1H), 7.32 (s, 1H), 7.52–7.57 (m, 4H), 9.68 (br, 1H), 10.05 (br, 1H); Anal. Calcd for C_20_H_18_O_5_: C, 71.00, H, 5.36. Found C, 70.91, H, 5.42.

#### 3.2.6. Synthesis of Compounds **6a,b**

To a solution of cyclohexanone (0.98 g, 10 mmol) in EtOH (40 mL), were added KOH (1.12 g, 20 mmol) and furfural (1.92 g, 20 mmol) or 2-thiophenecarboxaldehyde (2.24 g, 20 mmol). The mixture was stirred at r.t. for 2 h. The yellow precipitate was filtered and washed with water (× 3). The crude product was purified by recrystallization from EtOH.

(2*E*,6*E*)-2,6-bis (furan-2-ylmethylene)cyclohexan-1-one **6a** [48]
Orange needles, Yield: 2.26 g (89%); ^1^H NMR (500 MHz, DMSO-*d*_6_): δ 1.81 (quintet, *J* = 6.2 Hz, 2H), 2.95 (t, *J* = 6.2 Hz, 4H), 6.69 (dd, *J* = 1.8 Hz, *J* = 3.6 Hz, 2H), 6.95 (d, *J* = 3.6 Hz, 2H), 7.40 (s, 2H), 7.92 (d, *J* = 1.8 Hz, 2H); Anal. Calcd for C_16_H_14_O_3_: C, 75.58, H, 5.55. Found C, 75.68, H, 5.49.
(2*E*,6*E*)-2,6-bis (thiophen-2-ylmethylene)cyclohexan-1-one **6b** [48]
Yellow needles, Yield: 2.10 g (73%); ^1^H NMR (500 MHz, DMSO-*d*_6_): δ 1.90 (quintet, *J* = 6.2 Hz, 2H), 2.88 (t, *J* = 6.2 Hz, 4H), 7.15 (dd, *J* = 3.6 Hz, *J* = 5.0 Hz, 2H), 7.60 (d, *J* = 3.6 Hz, 2H), 7.86 (s, 2H), 7.90 (d, *J* = 5.0, 2H); Anal. Calcd for C_16_H_14_OS_2_: C, 67.10, H, 4.93, S, 22.39. Found C, 67.17, H, 4.99, S, 22.34.

#### 3.2.7. Synthesis of Compounds **6c–e**

To a solution of the appropriate aromatic aldehyde (20 mmol) in EtOH (10 mL), were added acetone (0.73 mL, 10 mmol) and a dispersion of NaOH (5 g, 125 mmol) in EtOH (40 mL). The mixture was stirred at r.t. for 3 h. Upon completion of the reaction, water (30 mL) was added and the precipitate was filtered, washed with water (× 3) and recrystallized from EtOH.

(1*E*,4*E*)-1,5-bis (4-methoxyphenyl) penta-1,4-dien-3-one **6c** [49]
Light yellow needles, Yield: 1.84 mg (63%); ^1^H NMR (500 MHz, DMSO-*d*_6_): δ; 3.82 (s, 6H), 7.02 (d, *J* = 8.6 Hz, 4H), 7.19 (d, *J* = 15.8 Hz, 2H), 7.73 (d, *J* = 15.8, 2H), 7.74 (d, *J* = 8.6 Hz, 4H); Anal. Calcd for C_19_H_18_O_3_: C, 77.53, H, 6.16. Found C, 77.45, H, 6.14.
(1*E*,4*E*)-1,5-bis (3,4-dimethoxyphenyl) penta-1,4-dien-3-one **6d** [50]
Yellow neeedles, Yield: 2.84 mg (72%); ^1^H NMR (500 MHz, DMSO-*d*_6_): δ 3.82 (s, 6H), 3.84 (s, 6H), 7.03 (d, *J* = 8.3 Hz, 2H), 7.23 (d, *J* = 15.9 Hz, 2H), 7.33 (d, *J* = 8.3 Hz, 2H), 7.41 (s, 2H), 7.70 (d, *J* = 15.9 Hz, 2H); Anal. Calcd for C_21_H_22_O_5_: C, 71.17, H, 6.26. Found C, 71.11, H, 6.31.
(1*E*,4*E*)-1,5-bis (3,4,5-trimethoxyphenyl) penta-1,4-dien-3-one **6e** [51]
Yellow needles, Yield: 1.92 mg (46%); ^1^H NMR (500 MHz, DMSO-*d*_6_): δ 3.71 (s, 6H), 3.85 (s, 12H), 7.13 (s, 4H), 7.31 (d, *J* = 16.0 Hz, 2H), 7.71 (d, *J* = 16.0 Hz, 4H); Anal. Calcd for C_23_H_26_O_7_: C, 66.65, H, 6.32. Found C, 66.71, H, 6.27.

#### 3.2.8. Synthesis of Compounds **6f–h**

To a solution of vanillin (3.04g, 20 mmol) or 3,4-dihydroxybenzaldehyde (2.76 g, 20 mmol) in EtOH (3 mL), were added acetone (0.72 mL, 10 mmol) or cyclopentanone (0.84 g and concentrated HCl (0.2 mL, 37%). The mixture was stirred at r.t. for 24 h. Upon completion of the rection, the solvent was concentrated to a volume of 0.5 mL approximately and the solution is added to ice-cold water. Aqueous KOH (1%) was added until pH 6–7 and the precipitate was filtered and washed with water (×2) and then with warm water (~60 °C). The crude product is purified by recrystallization from EtOH and water.

(1*E*,4*E*)-1,5-bis (4-hydroxy-3-methoxyphenyl) penta-1,4-dien-3-one **6f** [52]
Orange solid, Yield: 2.15 g (66%), ^1^H NMR (500 MHz, DMSO-*d*_6_): 3.85 (s, 6H), 6.83 (d, *J* = 8.2 Hz, 2H), 7.15 (d, *J* = 16.0 Hz, 2H), 7.20 (d, *J* = 8.2 Hz, 2H), 7.37 (s, 2H), 7.65 (d, *J* = 16.0 Hz, 2H), 9.65 (2H, s, OH); Anal. Calc. for C_19_H_18_O_5_: C 69.93, H 5.56. Found: C 69.82, H 5.50.
(1*E*,4*E*)-1,5-bis (3,4-dihydroxyphenyl) penta-1,4-dien-3-one **6g** [52]
Dark green solid, Yield: 1.45 g (49%); ^1^H NMR (500 MHz, DMSO-*d*_6_): δ 6.79 (d, *J* = 8.2 Hz, 2H), 6.99 (d, *J* = 16.0 Hz, 2H), 7.07 (d, *J* = 8.2 Hz, 2H), 7.14 (s, 2H), 7.56 (d, *J* = 16.0 Hz, 2H), 9.38 (br, 4H); Anal. Calcd for C_17_H_14_O_5_: C, 68.45, H, 4.73. Found C, 68.38, H, 4.79.
2,5-bis ((*E*)-3,4-dihydroxybenzylidene) cyclopentan-1-one **6h** [53]
Brown solid, Yield: 1.75 g (54%); ^1^H NMR (500 MHz, DMSO-*d*_6_): δ 3.00 (s, 4H), 6.83 (d, *J* = 8.2 Hz, 2H), 7.01 (d, *J* = 8.2 Hz, 2H), 7.11 (s, 2H), 7.24 (d, 2H), 9.39 (brs, 4H); Anal. Calcd for C_19_H_16_O_5_: C, 70.36, H, 4.97. Found C, 70.30, H, 5.06.

#### 3.2.9. Synthesis of Compound **6i**

Compound **6e** (207 mg, 0.5 mmol) was dissolved in anhydrous DCM (10 mL) in a 50 mL two-necked round bottom flask under N_2_. The solution was cooled to −20 °C using ice/NaCl bath. Boron tribromide (3.5 mmol) was injected carefully with a syringe. The reaction mixture was stirred for 1 h at −20 °C, then for 1 h at 0 °C and then for 1 h at rt. Upon completion of the reaction, ice-water mixture was poured into the reaction mixture and the flask was shaken for a few minutes. The precipitated was filtered and washed with small volumes of water (2 × 5 mL).

(1*E*,4*E*)-1,5-bis (3,4,5-trihydroxyphenyl) penta-1,4-dien-3-one **6i** [52]
Dark green solid, Yield: 74 mg (45%); ^1^H NMR (500 MHz, DMSO-*d*_6_): δ 6.69 (s, 4H), 6.91 (d, *J* = 8.4 Hz, 2H), 7.45 (d, *J* = 8.4 Hz, 2H), 8.86 (br, 2H), 9.09 (br, 4H); Anal. Calcd for C_17_H_14_O_7_: C, 61.82, H, 4.27. Found C, 61.75, H, 4.33.

#### 3.2.10. Synthesis of Compound **7a**

To a solution of the 3,4-dihydroxybenzaldehyde (1.38 g, 10 mmol) in EtOH (2 mL), were added acetone (10 mL) and concentrated HCl (1 mL, 37%). The mixture was stirred at r.t. for 24 h. Upon completion of the reaction, the solvent was concentrated to a volume of 0.5 mL approximately and the solution is added to a small volume of ice-cold water. Aqueous KOH (1%) was added until pH 6–7 and the precipitate was filtered and washed with small amounts of water (2 × 3 mL).

(*E*)-4-(3,4-dihydroxyphenyl) but-3-en-2-one **7a** [54]
Yellow solid, Yield: 1.18 g (66%); ^1^H NMR (500 MHz, DMSO-*d*_6_): δ 2.27 (s, 3H), 6.48 (d, *J* = 16.4 Hz, 1H), 6.77 (d, *J* = 8.2 Hz, 1H), 7.01 (dd, *J* = 1.5, Hz, *J* = 8.2 Hz, 1H), 7.06 (d, *J* = 1.5 Hz, 1H), 7.45 (d, *J* = 16.4 Hz, 1H), 9.37 (s, 1H, OH); Anal. Calcd for C_10_H_10_O_3_: C, 67.41, H, 5.66. Found C, 67.46, H, 5.60.

#### 3.2.11. Synthesis of Compound **7b**

A solution of 3,4,5-trimethoxybenzaldehyde (1.96 g, 10 mmol) and NaOH 1 M (1 mL, 1 mmol) in acetone (30 mL) is stirred at r.t. for 24 h. The solution was concentrated to a volume of 10 mL and water (30 mL0 was added). The formed brown oil was separated from the aqueous solution and after drying under vacuum, it was purified by flash chromatography (CHCl_3_). An appropriate quantity of the resulting off-white solid (118 mg, 0.5 mmol) was dissolved in anhydrous DCM (10 mL) in a 50 mL two-necked round bottom flask under N_2_. The solution was cooled to −20 °C using ice/NaCl bath. Boron tribromide (2.0 mmol) was injected carefully with a syringe. The reaction mixture was stirred for 1 h at −20 °C, then for 1 h at 0 °C and then for 1 h at rt. Upon completion of the reaction, ice-water mixture was poured into the reaction mixture and the flask was shaken for a few minutes. The crude product was extracted with EtOAc (×3). The organic solvent was evaporated under vacuum and the oily product was purified by flash chromatography (DCM/MeOH = 95:5).

(*E*)-4-(3,4,5-trihydroxyphenyl) but-3-en-2-one **7b** [52]
Yellow solid, Yield: 52 mg (54% calculated from 3,4,5-trimethoxy intermediate); ^1^H NMR (500 MHz, DMSO-*d*_6_): δ 2.27 (s, 3H), 6.38 (d, *J* = 16.2 Hz, 1H), 6.61 (s, 2H), 7.36 (d, *J* = 16.2 Hz, 1H), 8.95 (br, 3H); Anal. Calcd for C_10_H_10_O_4_: C, 61.85, H, 5.19. Found C, 61.89, H, 5.12.

### 3.3. Larvicidal Evaluation

#### 3.3.1. Mosquito Rearing

Mosquito larvae were obtained from laboratory colonies of *Ae. albopictus* and *Cx. p.* biotype *molestus*, which were maintained at 25 ± 2 °C, 80% relative humidity and photoperiod of LD 16:8h in the laboratory of Benaki Phytopathological Institute, Kifissia, Greece. Wood-framed cages (33 × 33 × 33 cm^3^) covered by a 32 × 32 mesh were used to keep adult mosquitoes of each species separately, ensuring easy access to 10% sucrose solution through a cotton wick. They have not been treated with any product prior to the tests to avoid establishment of resistance.

*Aedes albopictus* females were chicken blood fed by using the Hemotek membrane feeding system (Hemotek). Larvae were reared in tap water-filled cylindrical enamel pans and fed ad libitum with powdered fish food (JBL Novo Tom 10% Artemia) until the adults emerged. Plastic beakers with 100 mL water and strips of moistened filter paper were provided in the cage for oviposition. The eggs were kept wet for a few days and then placed in the pans for hatching.

*Culex pipiens* biotype *molestus* females were not blood-fed since this biotype is an autogenous biotype, i.e., female mosquitoes are able to produce their first egg-batch without a blood meal. Mosquito larvae were reared in tap-water-filled cylindrical enamel pans and were fed ad libitum with powdered fish food (JBL Novo Tom 10% Artemia). Cages containing *Cx. p.* biotype *molestus* adults were provided with containers filled with tap water for oviposition [55,56].

#### 3.3.2. Larvicidal Bioassays

The larval mortality bioassays were carried out according to the test method of larval susceptibility as suggested by the World Health Organization [56] with modifications. Stock solutions of 10% (*w*/*v*) in dimethyl sulfoxide (DMSO) were prepared for each testing material. Twenty late-third- to early-fourth-instar mosquito larvae of *Ae. albopictus* and *Cx. pipiens* were exposed to different doses of the tested materials, expressed as ppm (mg/L), under laboratory conditions. Four replicates per dose were made and treatments with only tap water and 2% water solution of DMSO were included in each bioassay as control. The number of dead larvae was recorded after 24 h of exposure and the respective mortality percentage calculated.

All compounds were initially tested at 20 ppm. For compounds exhibiting mortality ≥10% after 24 h, various concentrations were used for larvicidal activity assessment, leading to a dose-response curve to determine their respective LC_50_ and LC_90_ values. For compounds with mortality percentages <10% after 24 h, only the % mortality at 20 ppm was recorded [55,56,57].

#### 3.3.3. Data Analysis

Data obtained from each dose-larvicidal bioassay (total mortality per milligram per liter of concentration in water) were subjected to probit analysis in which probit-transformed mortality was regressed against log10-transformed dose; LC_50_, LC_90_ values and slopes were generated. All analyses were conducted using the statistical package SPSS 14.0 [58].

### 3.4. In Silico Molecular Properties and Descriptors Predictions

The cheminformatics program Canvas [59] of Schrödinger software was utilized to generate molecular descriptors and properties for the synthesized compounds. Particularly, Canvas computes a wide range of properties, including Topological, Physicochemical and Ligfilter descriptors. In total, more than 100 descriptors were calculated for all the examined compounds. Their values were used in an effort to compare the relationship between their experimental repellent activity and their descriptors profile. For this scope, all synthesized compounds were sketched in 2D form and then were subjected to energy minimization with OPLS2005 force field, using MacroModel program [60]. Minimization was performed with Powell-Reeves Conjugate Gradient (PRCG) method, using 1000 iterations and convergence threshold of 0.001 kcal/mol Å.

## Figures and Tables

**Table 1:** LC_50_ and LC_90_ values of four compounds against the third- to fourth-instar larvae of *Aedes albopictus* and *Culex pipiens* biotype *molestus* after 24 h exposure (SE: standard error; d.f.: degrees of freedom).

	Compound	Slope (±SE)	LC_50_ (95% CL) ^a^	LC_90_ (95% CL) ^a^	x^2^	df
*Aedes albopictus*	**1f**	6.4 ± 0.6	9.2 8.5–9.9	14.5 12.8–17.8	37.064	20
	**5**	9.7 ± 1.1	36.0 32.6–39.5	48.8 43.6–60.9	26.174 ^b^	10
	**3f**	2.3 ± 0.2	5.5 4.6–6.3	20.2 16.4–26.9	14.302	22
	**6d**	4.2 ± 0.5	23.6 21.3–26.2	47.38 40.5–59.8	14.013	10
*Culex pipiens*	**1f**	4.0 ± 0.4	6.0 5.4–6.6	12.5 11.0–15.0	10.395	14
	**5**	3.1 ± 0.3	9.4 8.2–10.6	24.0 20.2–30.5	10.194 ^b^	13
	**3f**	2.4 ± 0.2	5.0 4.4–5.7	17.0 14.3–21.2	15.977	24
	**6d**	10.6 ± 0.9.	32.5 31.4–33.7	42.93 40.8–45.9	12.193	17

^a^ LC values are expressed in ppm (mg/L) and they are considered significantly different when 95% CL fail to overlap; ^b^ Since goodness–of–fit test is significant (*p* < 0.05), a heterogeneity factor is used in the calculation of confidence limits (CL).

**Table 2:** Predictive physicochemical descriptors values of the tested compounds.

Compound	AlogP	HBA	HBD	RB	PSA	Estate	MR	Polar
1a	3.54	4	0	8	52.6	60.50	99.85	44.20
1b	3.51	6	0	10	71.06	70.84	112.77	49.14
1c	3.48	8	0	12	89.52	81.17	125.70	54.09
1d	3.04	4	2	6	74.6	61.50	90.31	40.53
1e	2.51	6	4	6	115.06	72.84	93.69	41.80
1f	**3.01**	**6**	**2**	**8**	**93.06**	**71.84**	**103.2**	**45.47**
1g	3.90	2	0	8	40.62	61.50	115.78	49.30
1h	4.51	2	0	8	34.14	58.17	107.56	46.21
1i	3.94	6	2	10	93.06	79.84	123.87	52.43
3f	**4.53**	**6**	**2**	**6**	**85.22**	**78.84**	**107.61**	**45.74**
3g	5.42	2	0	6	32.78	68.50	120.15	49.57
3h	6.03	2	0	6	26.3	65.17	111.93	46.48
3i	5.46	6	2	8	85.22	86.87	128.25	52.70
2	3.85	5	3	6	87.6	63.84	106.70	48.32
4	1.27	4	1	5	63.6	47.33	64.66	27.80
5	**3.03**	**5**	**2**	**7**	**83.83**	**66.67**	**96.77**	**43.00**
6a	3.12	3	0	2	43.35	42.83	69.08	31.65
6b	4.24	1	0	2	73.55	39.50	81.97	37.10
6c	3.77	3	0	6	35.53	50.33	89.95	40.44
6d	**3.74**	**5**	**0**	**8**	**53.99**	**60.67**	**102.88**	**45.39**
6e	3.71	7	0	10	72.45	71.00	115.81	50.33
6f	3.24	5	2	6	75.99	61.67	93.34	41.72
6g	2.74	5	4	4	97.99	66.67	83.80	38.05
6h	3.47	5	4	2	97.99	65.00	90.72	40.94
6i	2.20	7	6	4	138.45	74.00	87.19	39.32
7a	1.25	3	2	2	57.53	37.67	49.99	22.21
7b	0.98	4	3	2	77.76	43.33	51.69	22.84

Note: ALogP = logarithm of the octanol-water partition coefficient, HBA = number of hydrogen bond acceptors, HBD = number of hydrogen bond donors, RB = number of rotatable bonds PSA = Polar Surface Area, E-state = Electrotopological state, MR = Molar Refractivity, Polar = Molecular Polarizability.

## Data Availability

The data presented in this study are available in the supplementary information file.

## Supplementary Materials

Supplementary Materials can be found at https://www.mdpi.com/article/10.3390/ijms22168915/s1.
