# Supplementary material for: Curcumin Derivatives as Potential Mosquito Larvicidal Agents against Two Mosquito Vectors, Culex pipiens and Aedes albopictus
Source: Int J Mol Sci. 2021 Aug 18;22(16):8915. doi: 10.3390/ijms22168915 (PMC8396198; doi:10.3390/ijms22168915)
Supplement: Supplementary file 1 [file ijms-22-08915-s001.zip › ijms-1290690-supplementary.pdf]

Curcumin Derivatives as Potential Mosquito Larvicidal Agents against Two Mosquito Vectors, *Culex pipiens* and *Aedes albopictus*

Dimitris Matiadis [1], Panagiota G. V. Liggri [2,3], Eftichia Kritsi [4,5], Niki Tzioumaki [2,3], Panagiotis Zoumpoulakis [4,5], Dimitrios P. Papachristos [3], George Balatsos [3], Marina Sagnou [1,*] and Antonios Michaelakis [3,*]

[1] Institute of Biosciences and Applications, National Centre for Scientific Research "Demokritos", 153 10 Athens, Greece; matiadis@bio.demokritos.gr
[2] Department of Biochemistry and Biotechnology, University of Thessaly, Biopolis, 41500 Larissa, Greece
[3] Benaki Phytopathological Institute, Scientific Directorate of Entomology and Agricultural Zoology, 14561 Kifissia, Greece; p.liggri@bpi.gr (P.G.V.L.); nikitzioumaki@gmail.com (N.T.); d.papachristos@bpi.gr (D.P.P.); g.balatsos@bpi.gr (G.B.)
[4] Institute of Chemical Biology, National Hellenic Research Foundation, 48 Vas. Constantinou Avenue, 11635 Athens, Greece; ekritsi@eie.gr (E.K.); pzoump@uniwa.gr (P.Z.)
[5] Department of Food Science and Technology, University of West Attica, Ag. Spyridonos, 12243 Egaleo, Greece

*Correspondence: sagnou@bio.demokritos.gr (MS), a.michaelakis@bpi.gr (AM

Supporting Information

Contents

NMR spectra 2

NMR spectra

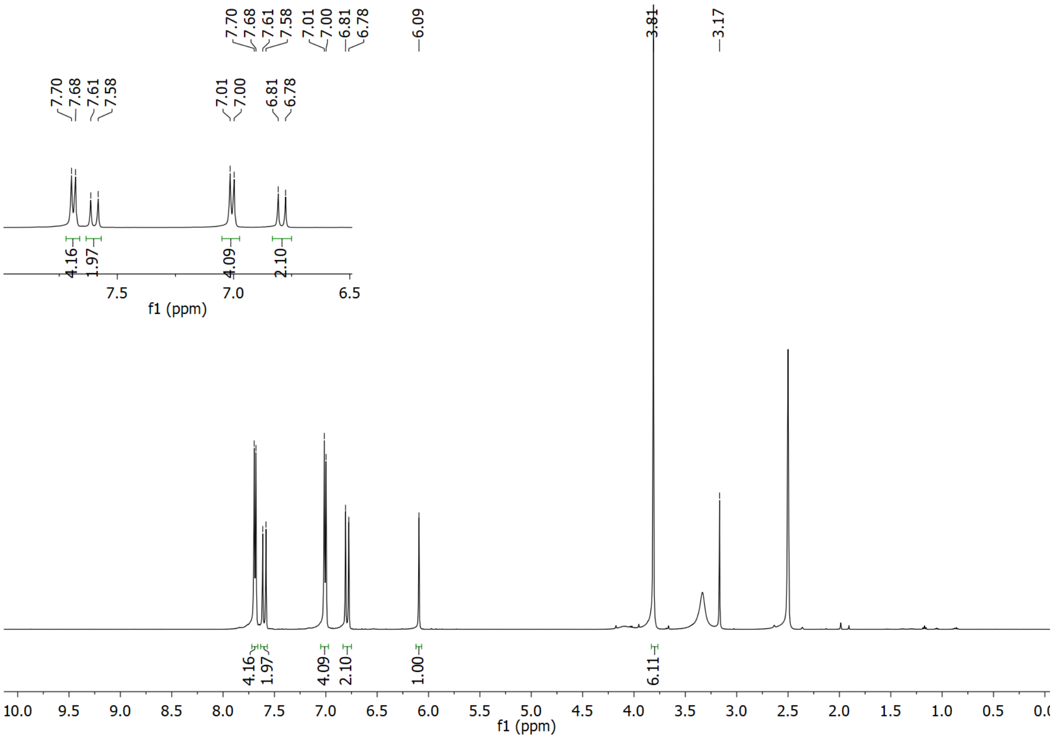

Figure S1. ^{1}H NMR spectrum of compound (1E,6E)-1,7-bis(4-methoxyphenyl)hepta-1,6-diene-3,5-dione **1a** in DMSO-d_6.

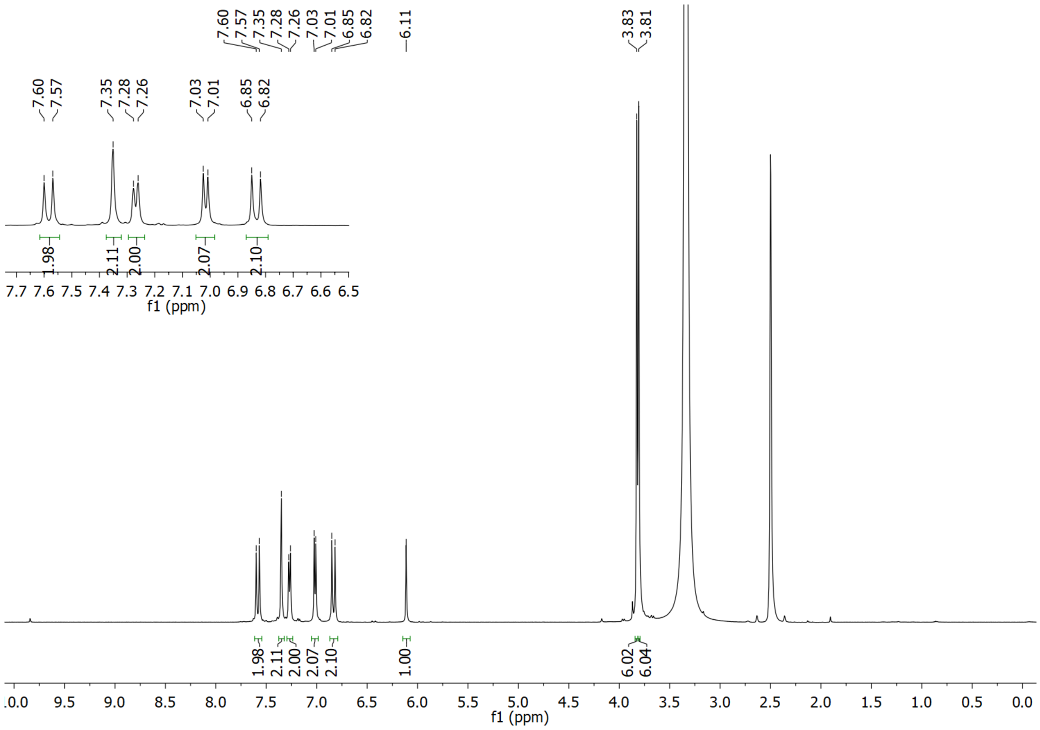

Figure S2. ^{1}H NMR spectrum of compound (1*E*,6*E*)-1,7-bis(3,4-dimethoxyphenyl)hepta-1,6-diene-3,5-dione **1b** in DMSO-d_6.

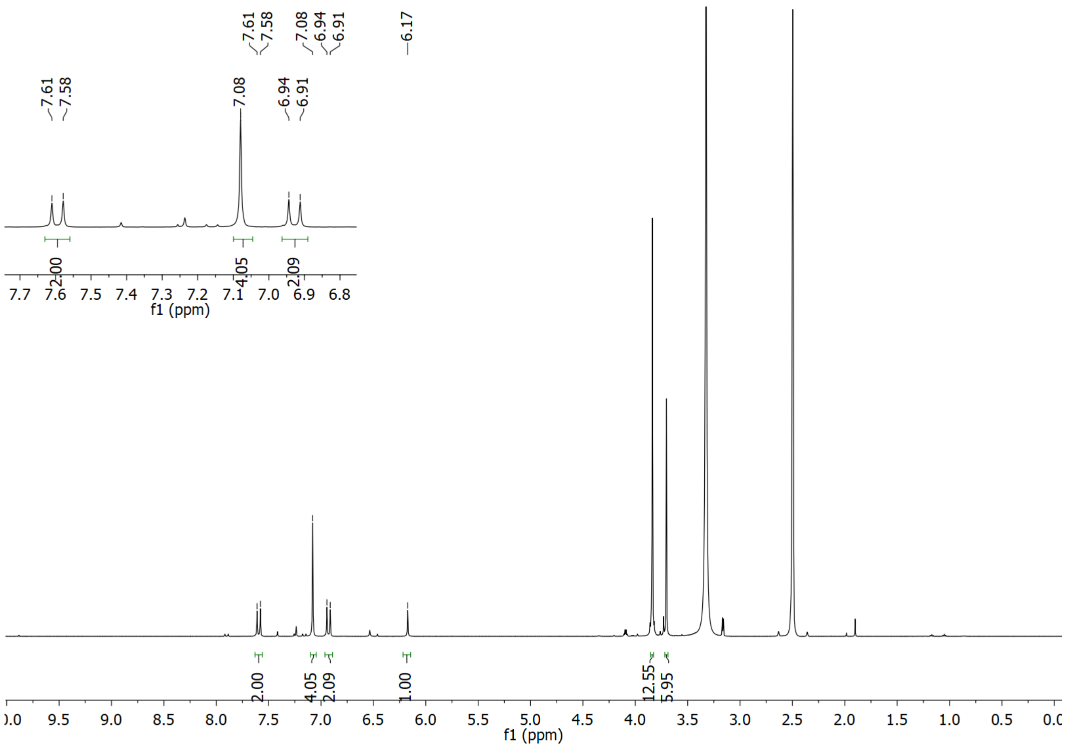

Figure S3. ^{1}H NMR spectrum of compound (1*E*,6*E*)-1,7-bis(3,4,5-trimethoxyphenyl)hepta-1,6-diene-3,5-dione **1c** in DMSO-d_6.

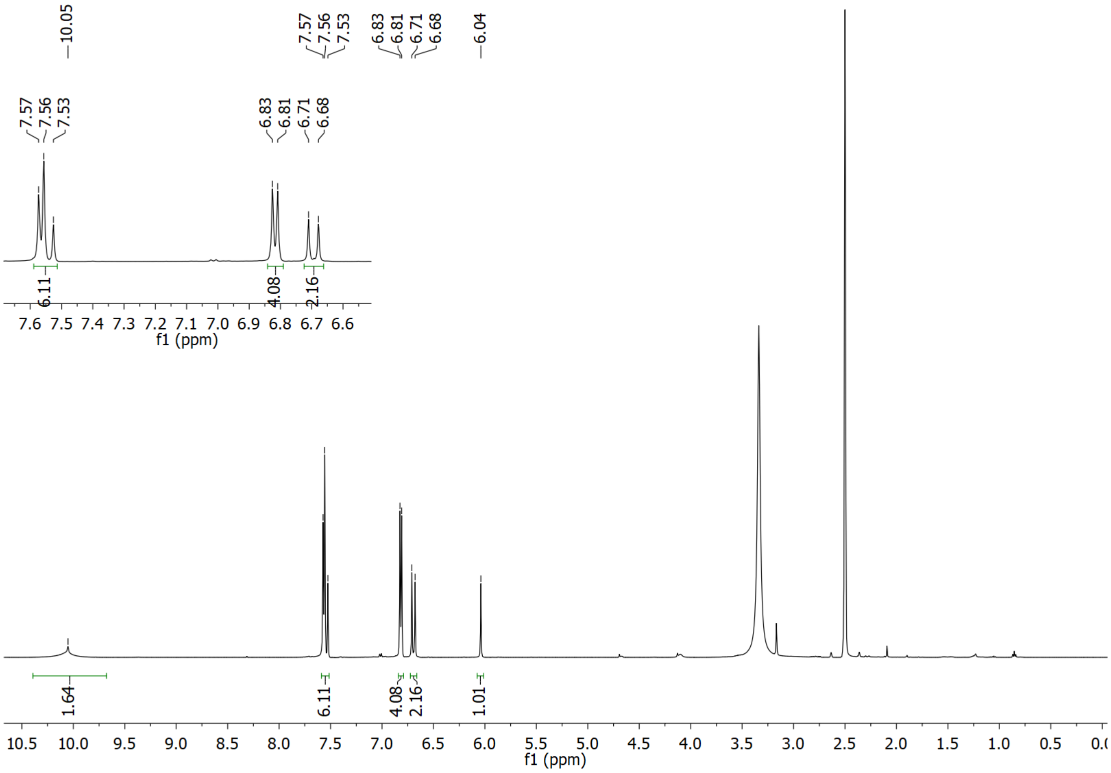

Figure S4. ^{1}H NMR spectrum of compound (1*E*,6*E*)-1,7-bis(4-hydroxyphenyl)hepta-1,6-diene-3,5-dione **1d** in DMSO-d_6.

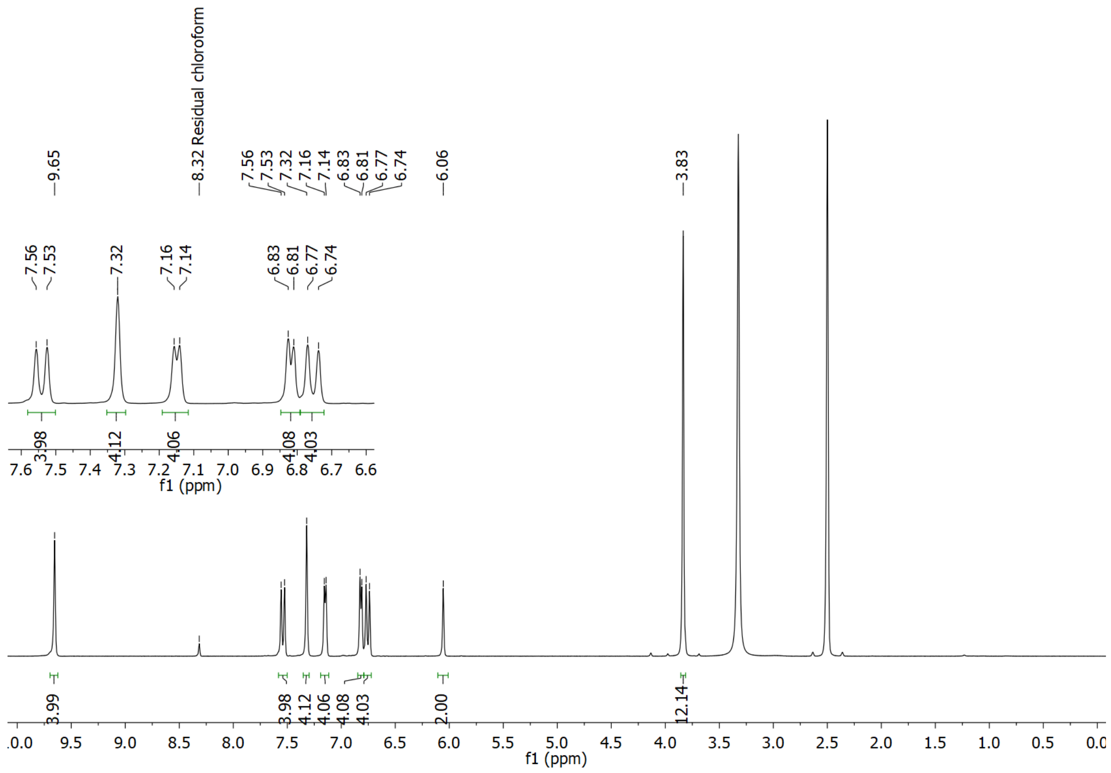

Figure S5. ^{1}H NMR spectrum of compound (1*E*,6*E*)-1,7-bis(4-hydroxy-3-methoxyphenyl)hepta-1,6-diene-3,5-dione **1f** in DMSO-d_6.

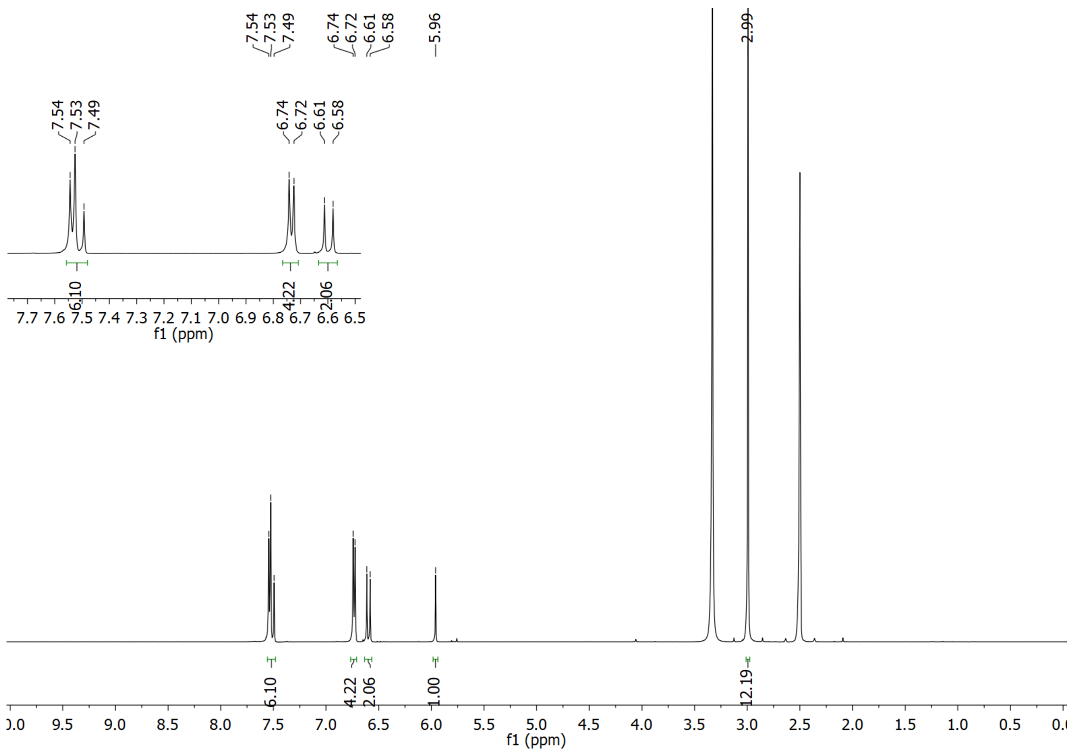

Figure S6. ^{1}H NMR spectrum of compound (1*E*,6*E*)-1,7-bis(4-(dimethylamino)phenyl)hepta-1,6-diene-3,5-dione **1g** in DMSO-d_6.

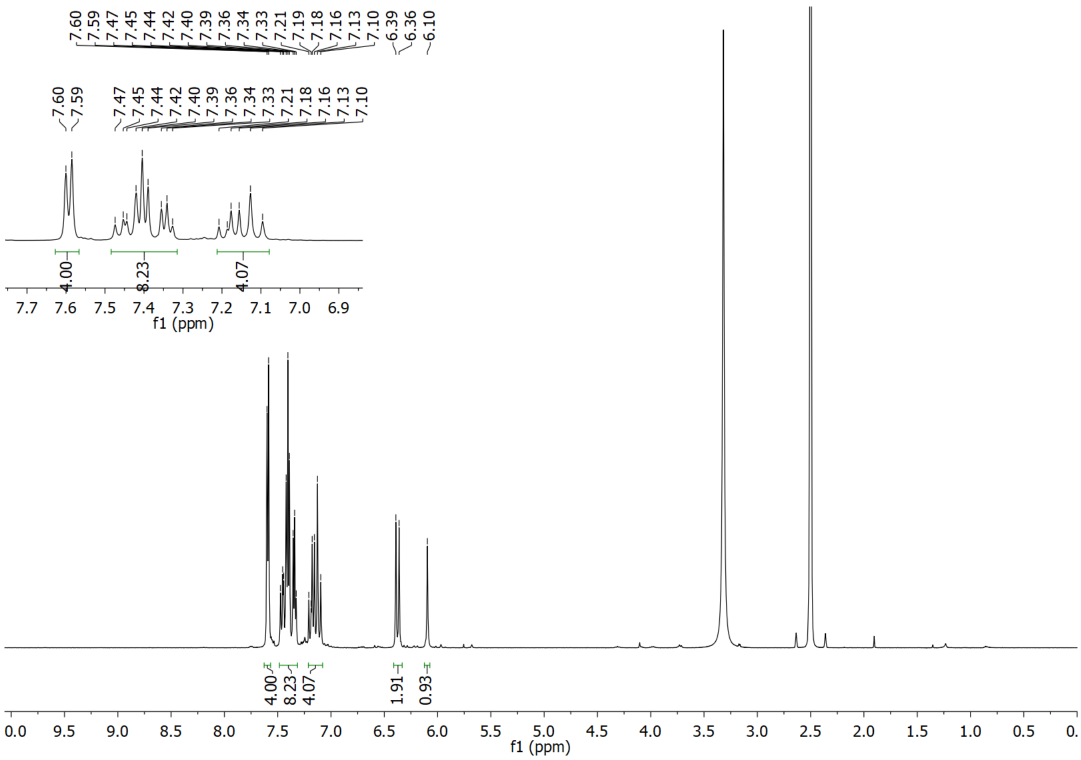

Figure S7. ^{1}H NMR spectrum of compound (1*E*,3*E*,8*E*,10*E*)-1,11-diphenylundeca-1,3,8,10-tetraene-5,7-dione **1h** in DMSO-d_6.

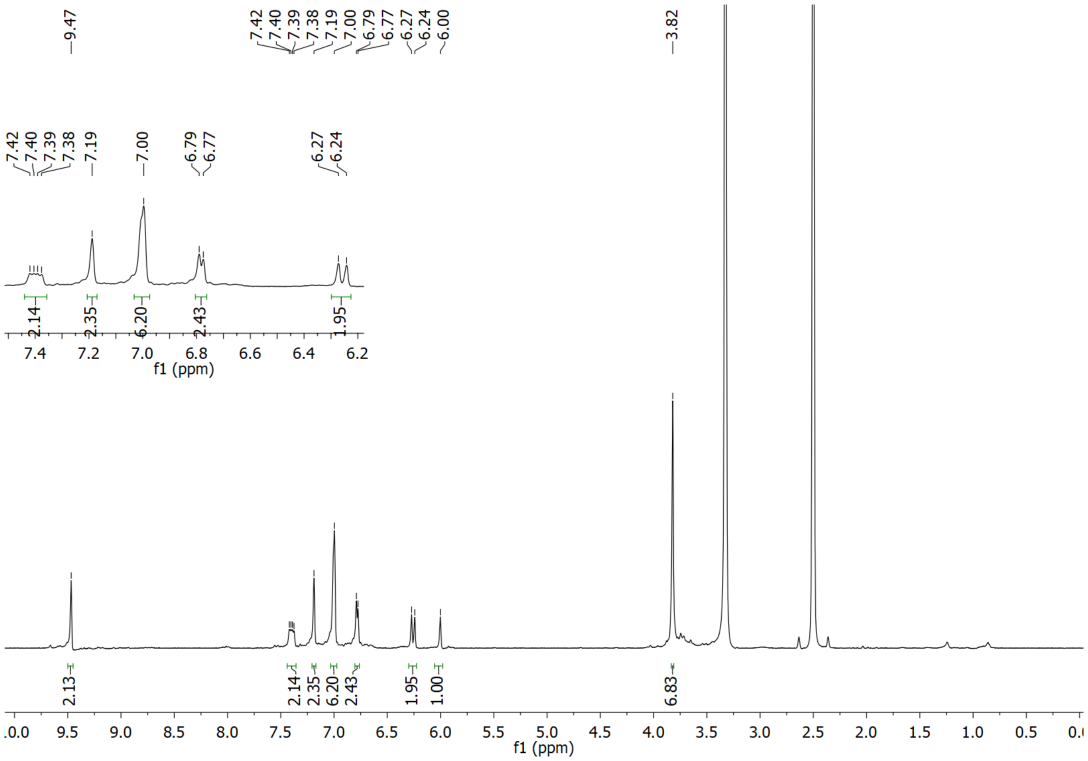

Figure S8. ^{1}H NMR spectrum of compound (1*E*,3*E*,8*E*,10*E*)-1,11-bis(4-hydroxy-3-methoxyphenyl)undeca-1,3,8,10-tetraene-5,7-dione **1i** in DMSO-d_6.

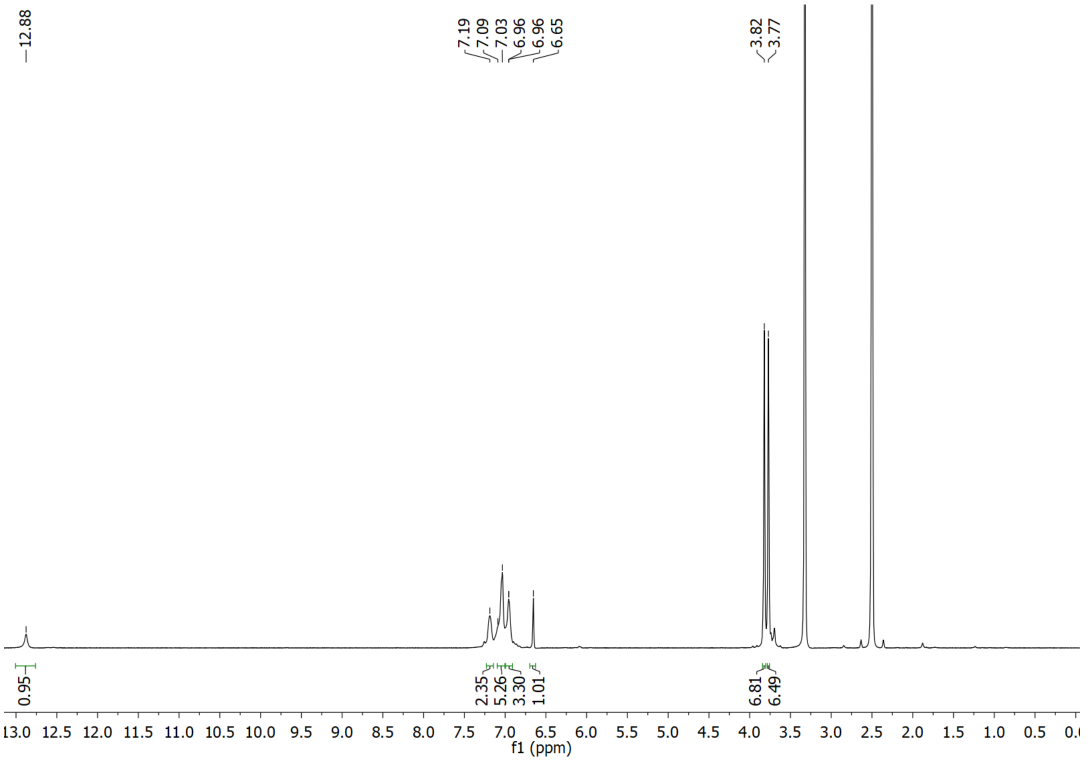

Figure S9. ^{1}H NMR spectrum of compound 4,4'-((1*E*,1'*E*)-(1*H*-pyrazole-3,5-diyl)bis(ethene-2,1-diyl))bis(2-methoxyphenol) **2** in DMSO-d_6.

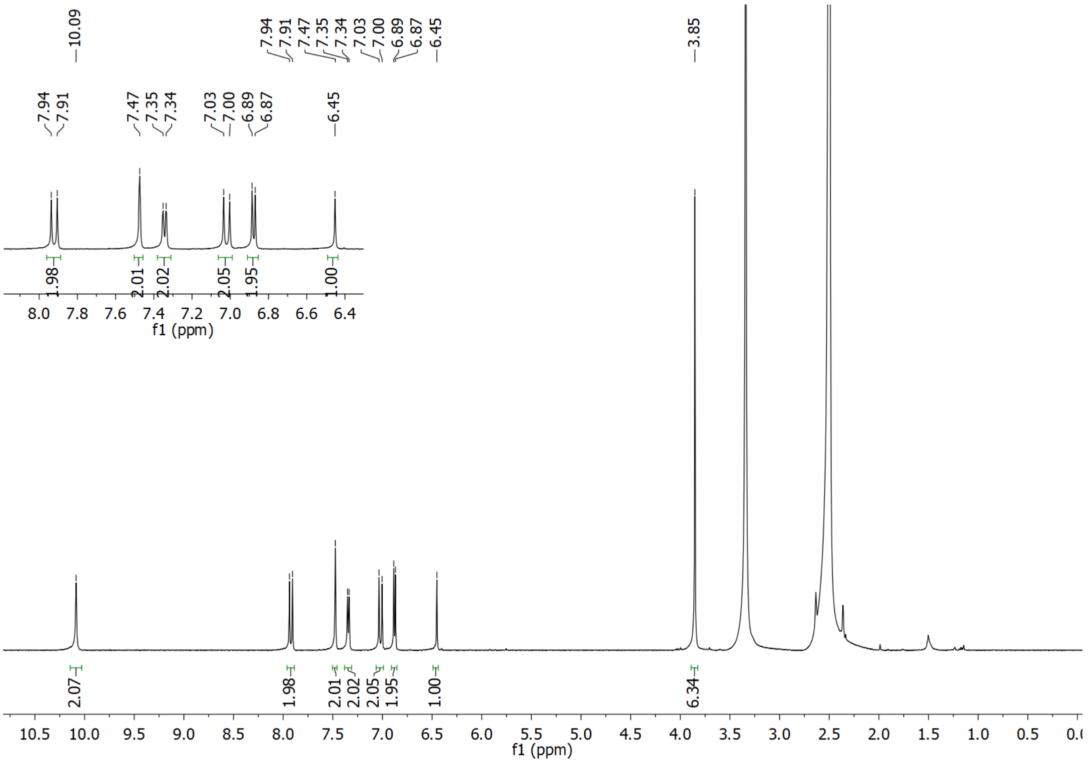

Figure S10. ^{1}H NMR spectrum of compound (1*E*,6*E*)-1,7-bis(4-hydroxy-3-methoxyphenyl)hepta-1,6-diene-3,5-dione-BF_2 complex **3f** in DMSO-d_6.

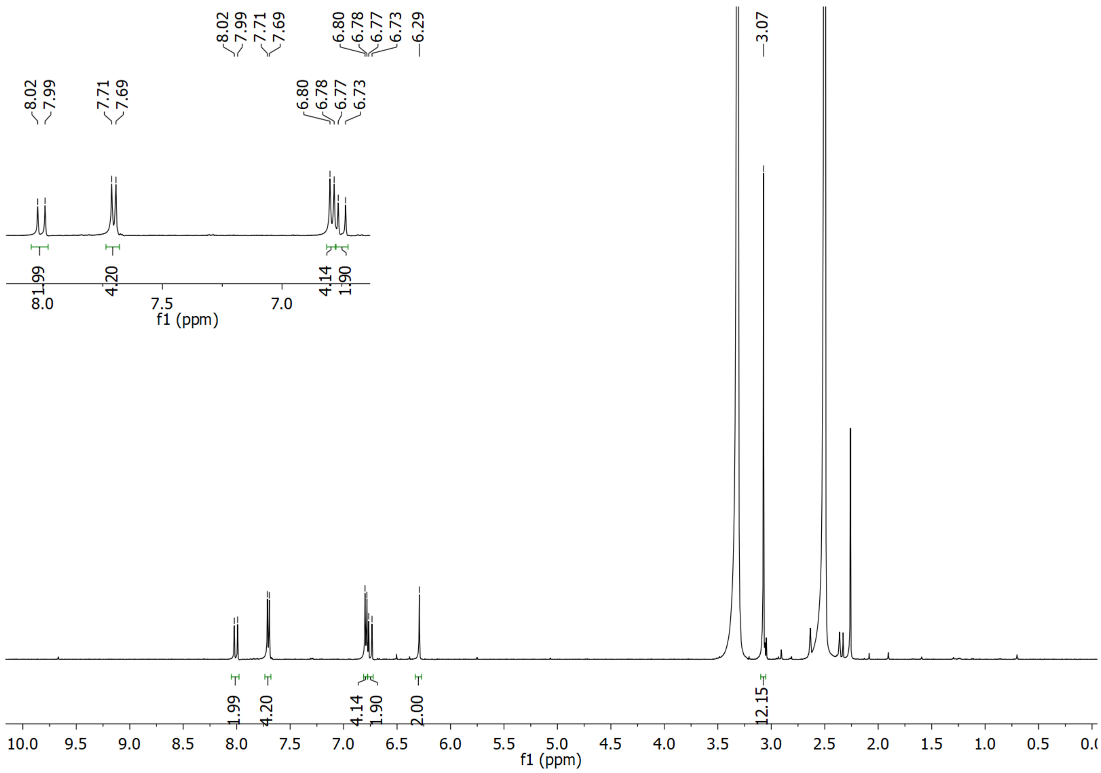

Figure S11. ^{1}H NMR spectrum of compound (1E,6E)-1,7-bis(4-(dimethylamino)phenyl)hepta-1,6-diene-3,5-dione-BF_2 complex **3g** in DMSO-d_6.

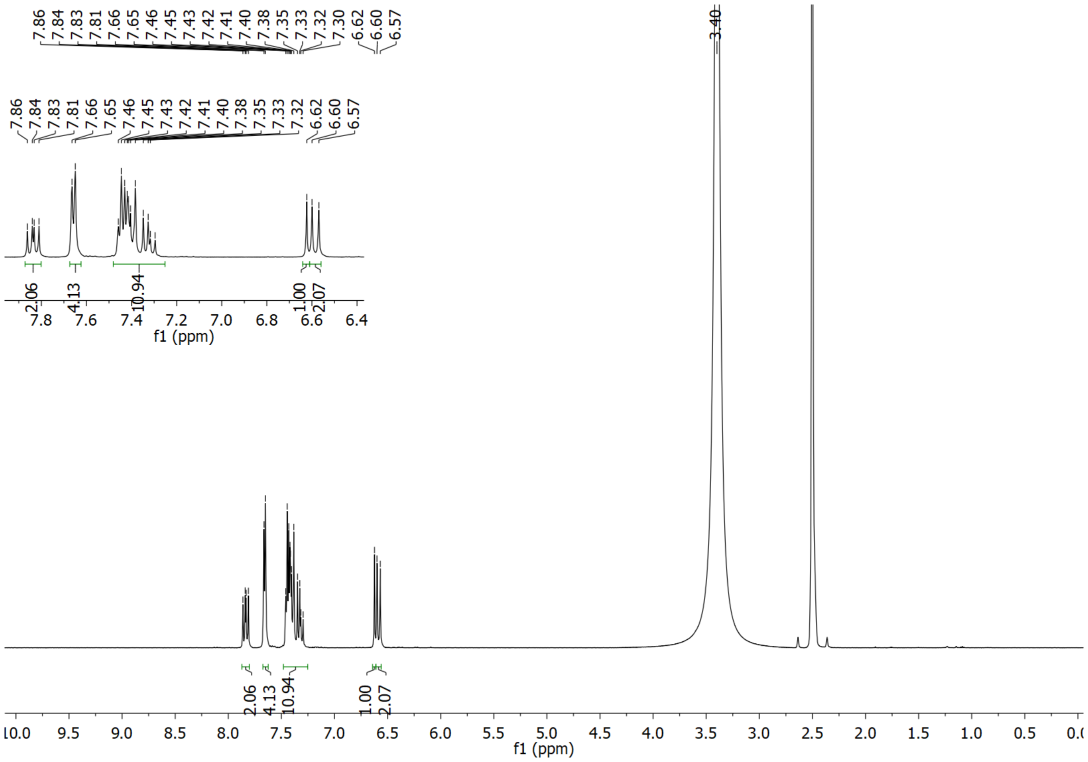

Figure S12. ^{1}H NMR spectrum of compound (1E,3E,8E,10E)-1,11-diphenylundeca-1,3,8,10-tetraene-5,7-dione-BF_2 complex **3h** in DMSO-d_6.

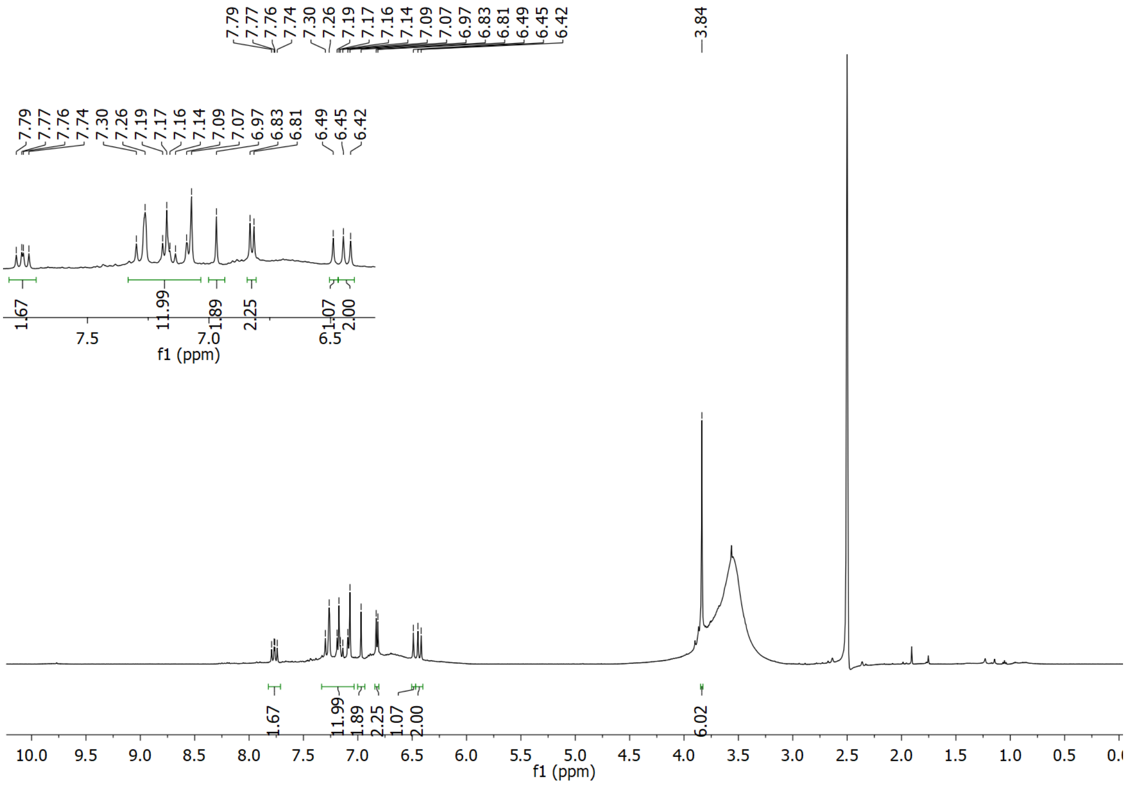

Figure S13. ^{1}H NMR spectrum of compound (1*E*,3*E*,8*E*,10*E*)-1,11-bis(4-hydroxy-3-methoxyphenyl)undeca-1,3,8,10-tetraene-5,7-dione-BF_2 complex **3i** in DMSO-d_6.

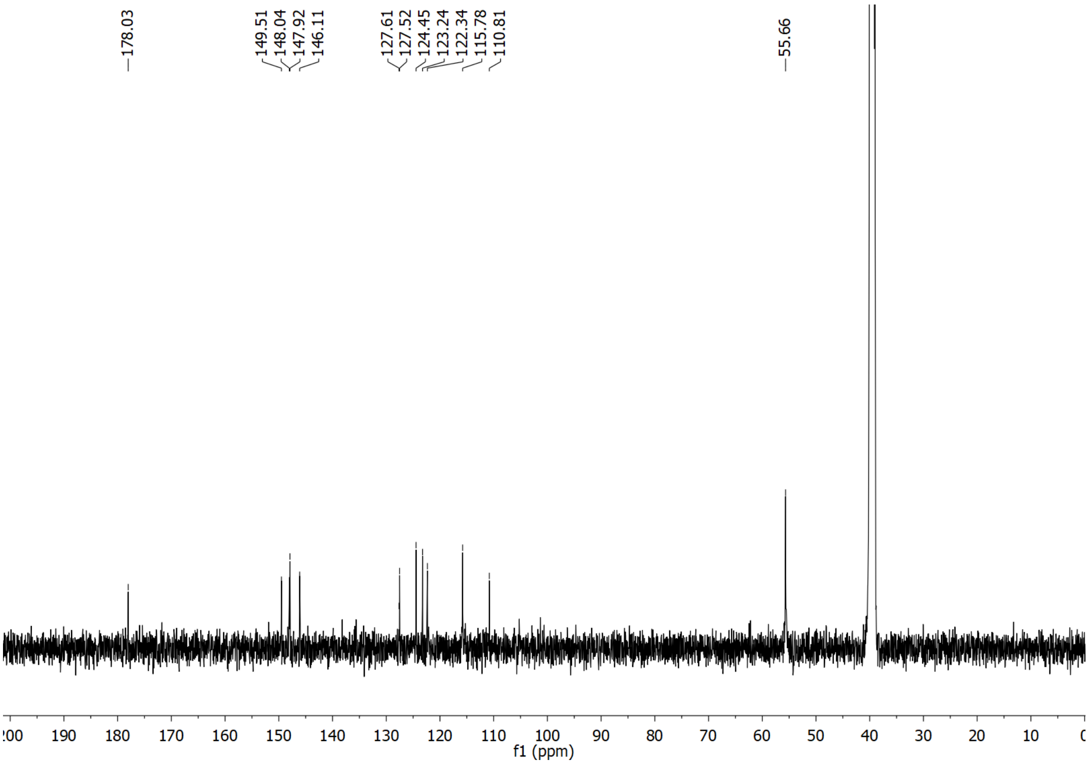

Figure S14. ^{13}C NMR spectrum of compound **3i** in DMSO-d_6.

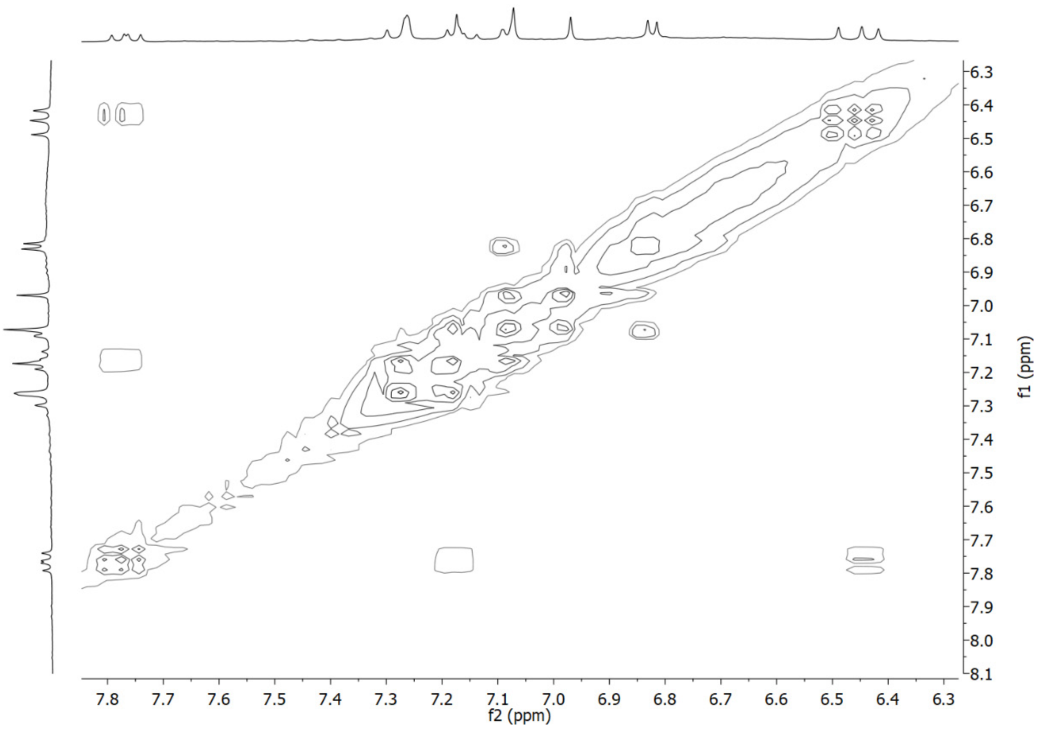

Figure S15. ^{1}H-^{1}H COSY spectrum of compound **3i** in DMSO-d_6.

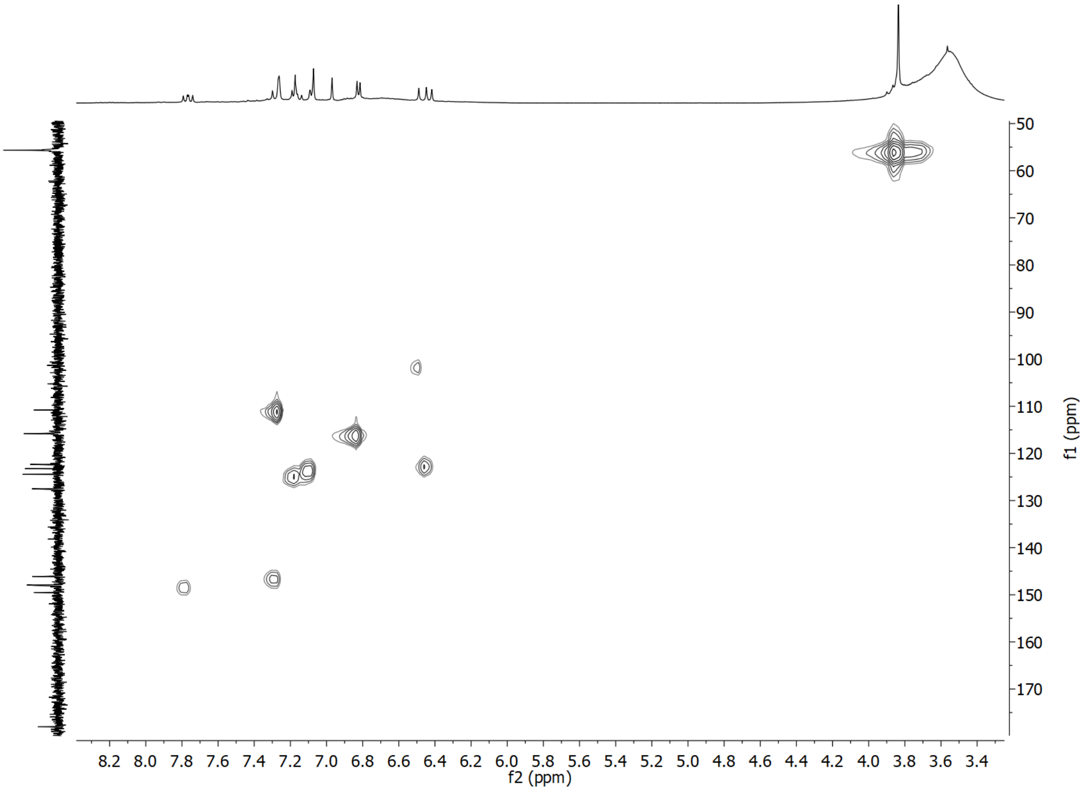

Figure S16. ^{1}H-^{13}C HSQC spectrum of compound **3i** in DMSO-d_6.

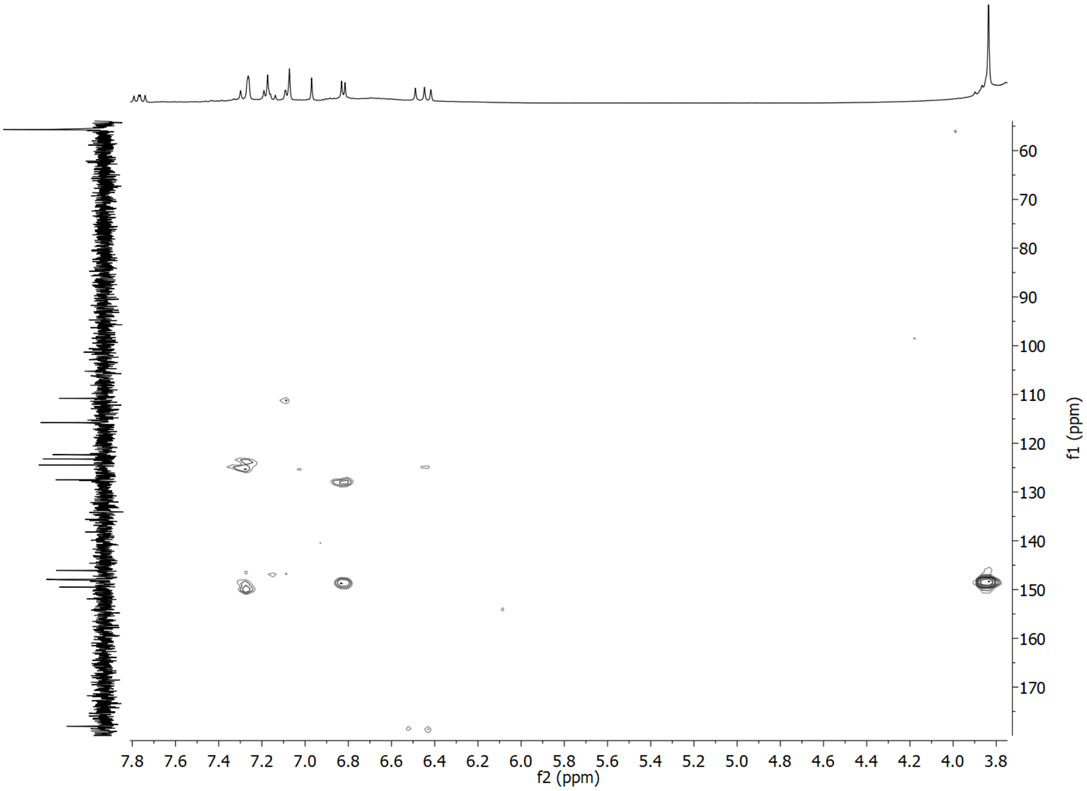

Figure S17. ^{1}H-^{13}C HMBC spectrum of compound **3i** in DMSO-d_6.

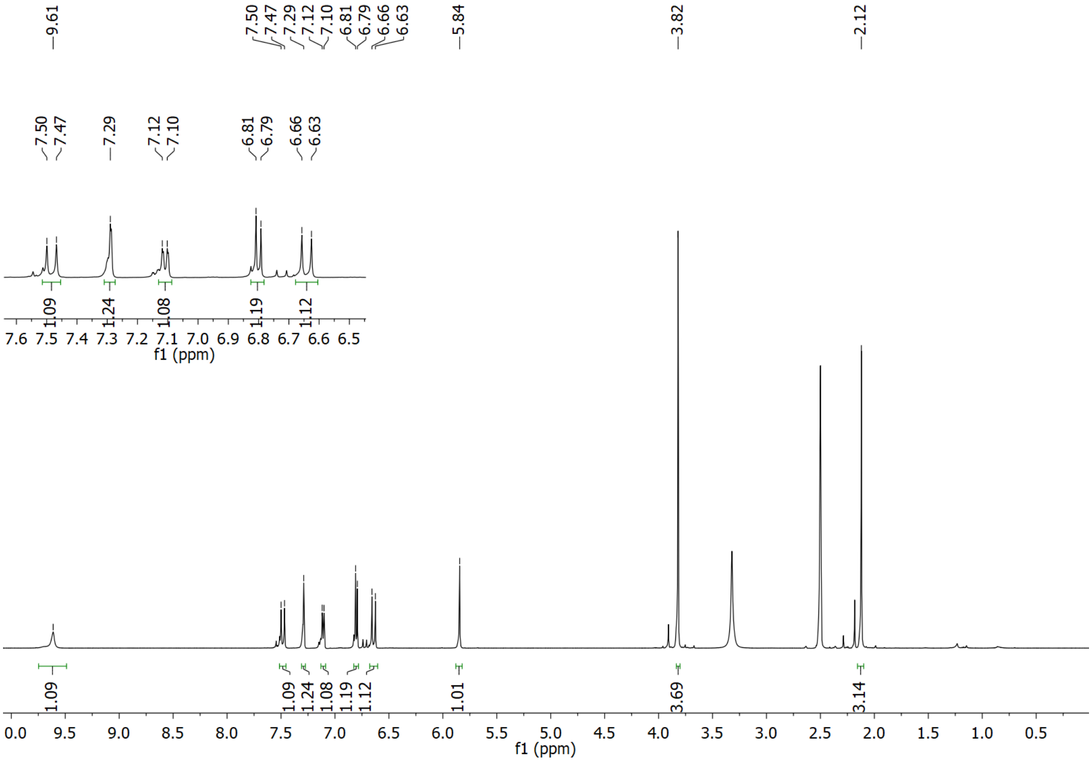

Figure S18. ^{1}H NMR spectrum of compound (*E*)-6-(4-hydroxy-3-methoxyphenyl)hex-5-ene-2,4-dione **4** in DMSO-d_6.

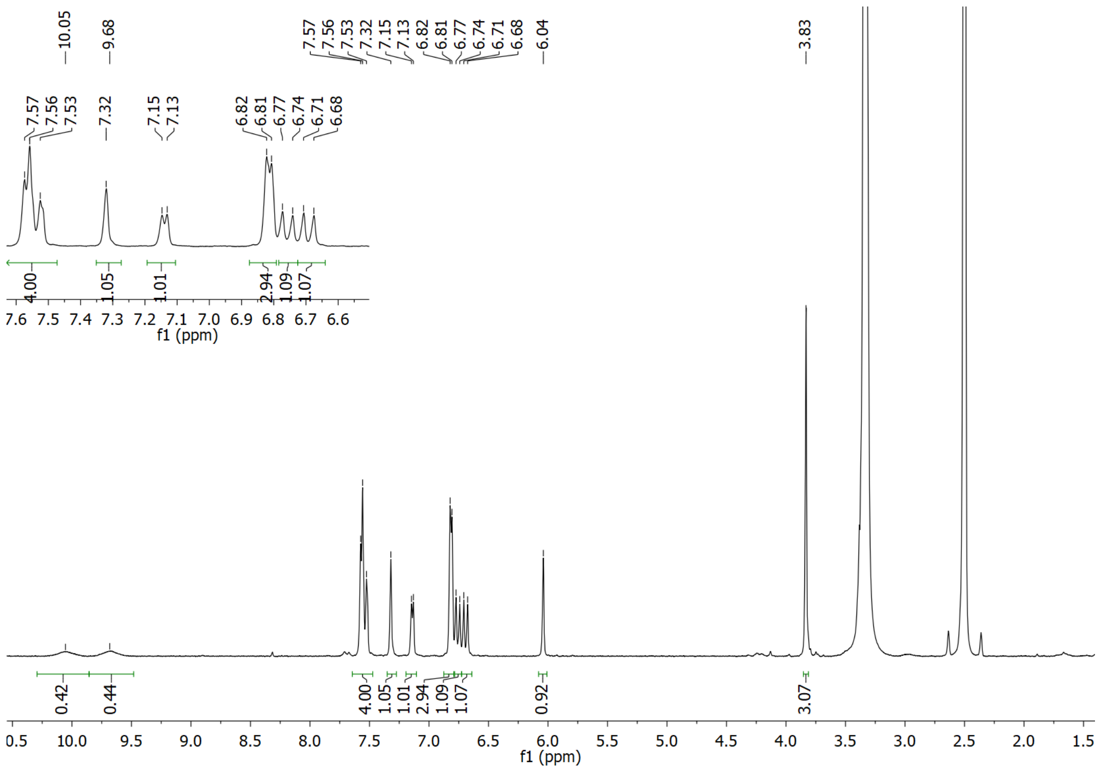

Figure S19. ^{1}H NMR spectrum of compound (1*E*,6*E*)-1-(4-hydroxy-3-methoxyphenyl)-7-(4-hydroxyphenyl)hepta-1,6-diene-3,5-dione **5** in DMSO-d_6.

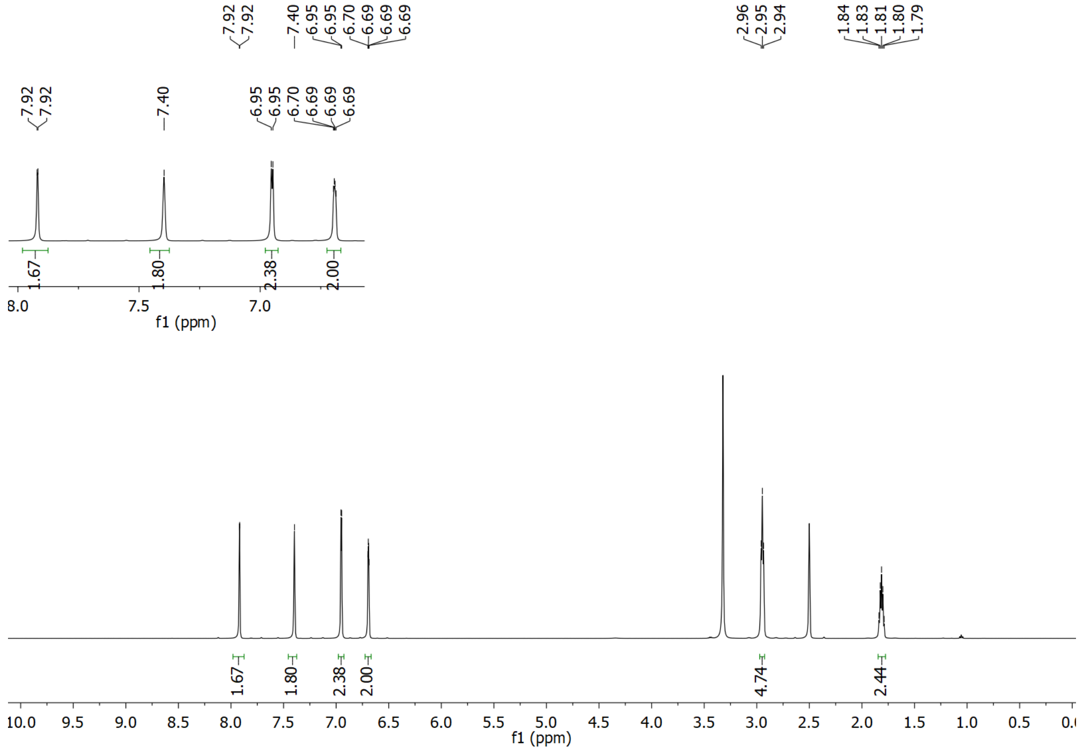

Figure S20. ^{1}H NMR spectrum of compound (2*E*,6*E*)-2,6-bis(furan-2-ylmethylene)cyclohexan-1-one **6a** in DMSO-d_6.

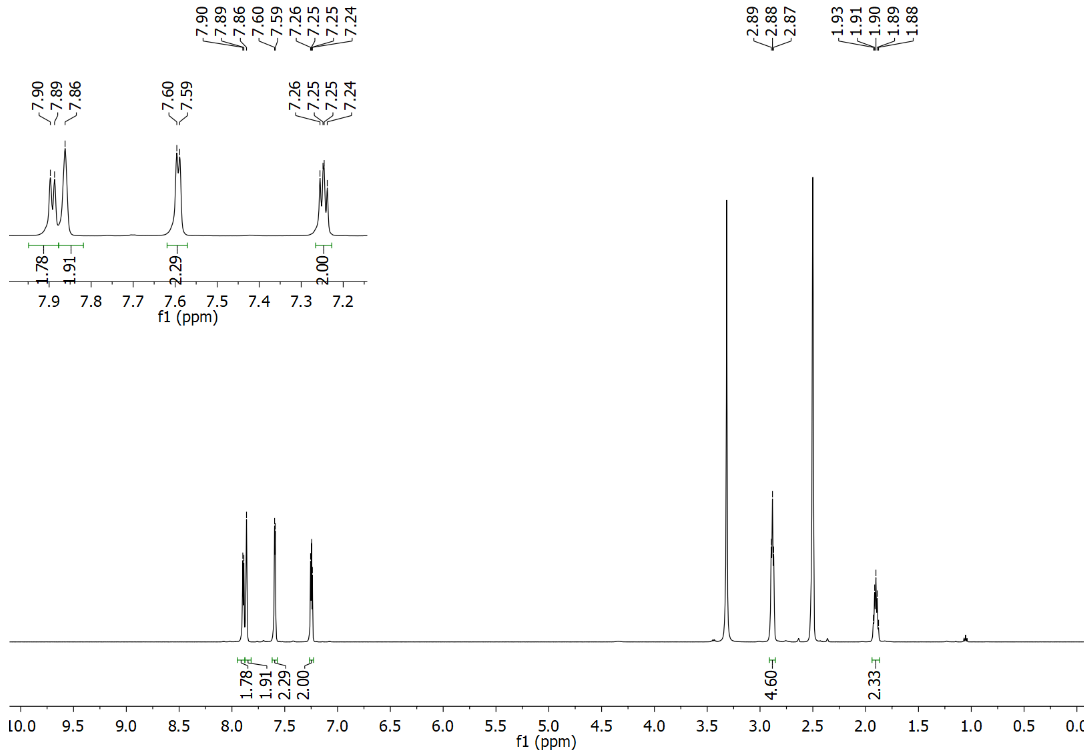

Figure S21. ^{1}H NMR spectrum of compound (2*E*,6*E*)-2,6-bis(thiophen-2-ylmethylene)cyclohexan-1-one **6b** in DMSO-d_6.

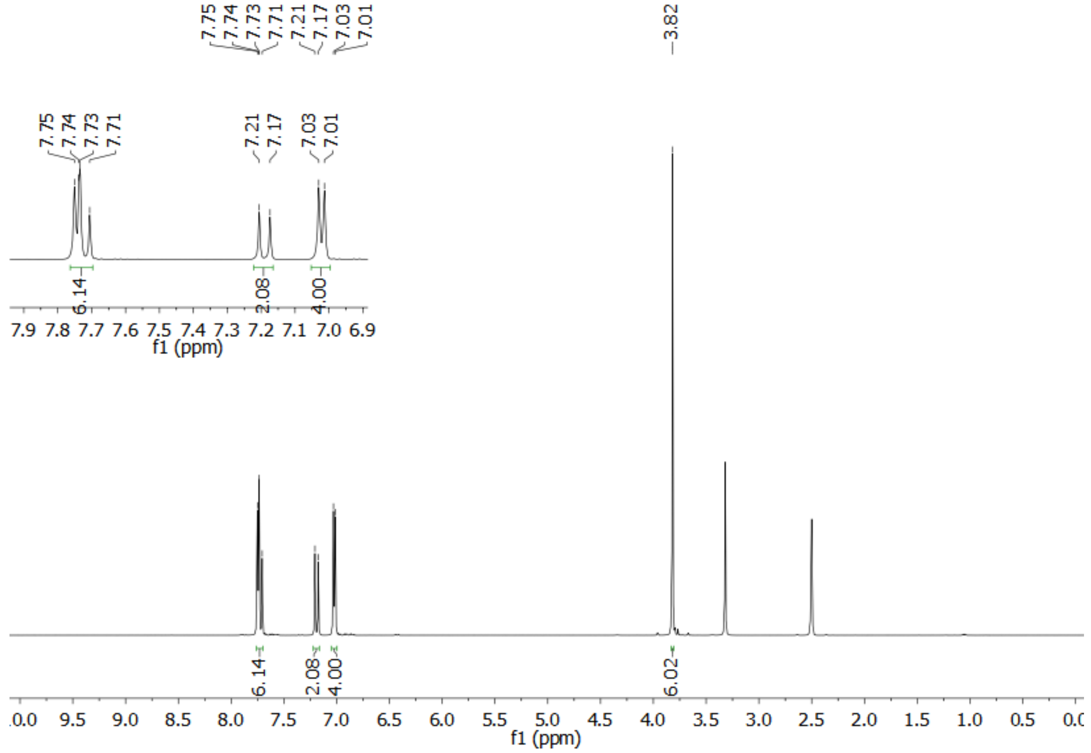

Figure S22. ^{1}H NMR spectrum of compound (1*E*,4*E*)-1,5-bis(4-methoxyphenyl)penta-1,4-dien-3-one **6c** in DMSO-d_6.

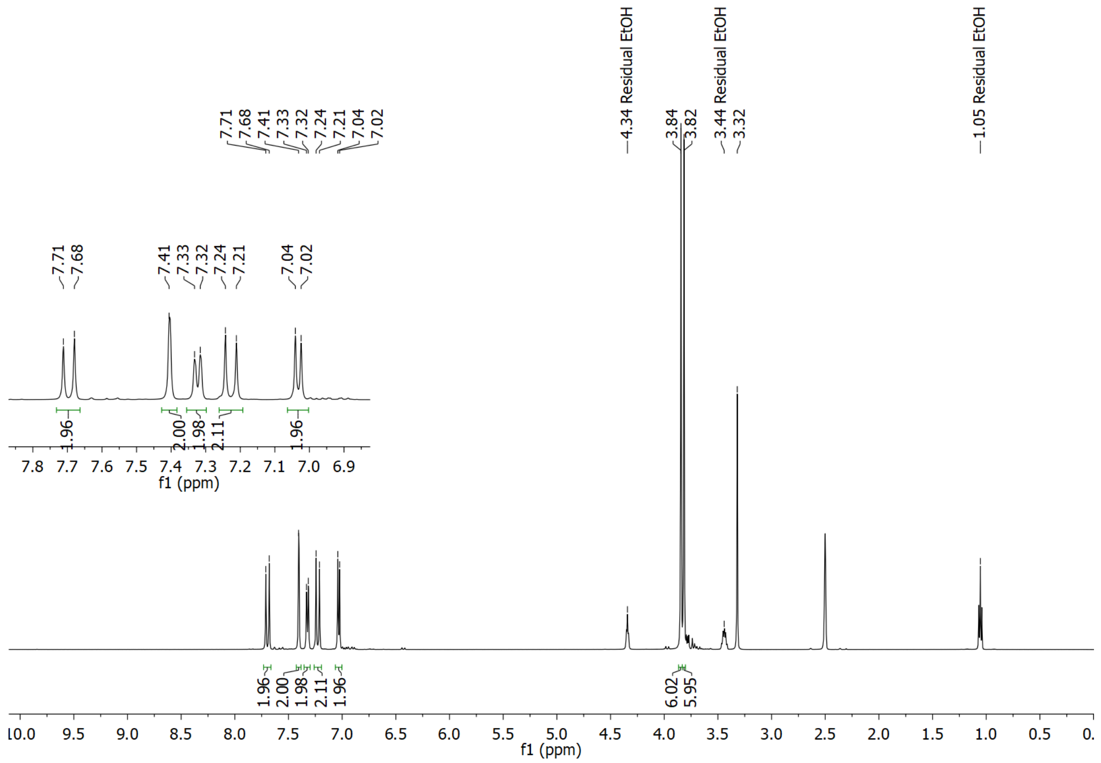

Figure S23. ^{1}H NMR spectrum of compound (1*E*,4*E*)-1,5-bis(3,4-dimethoxyphenyl)penta-1,4-dien-3-one **6d** in DMSO-d_6.

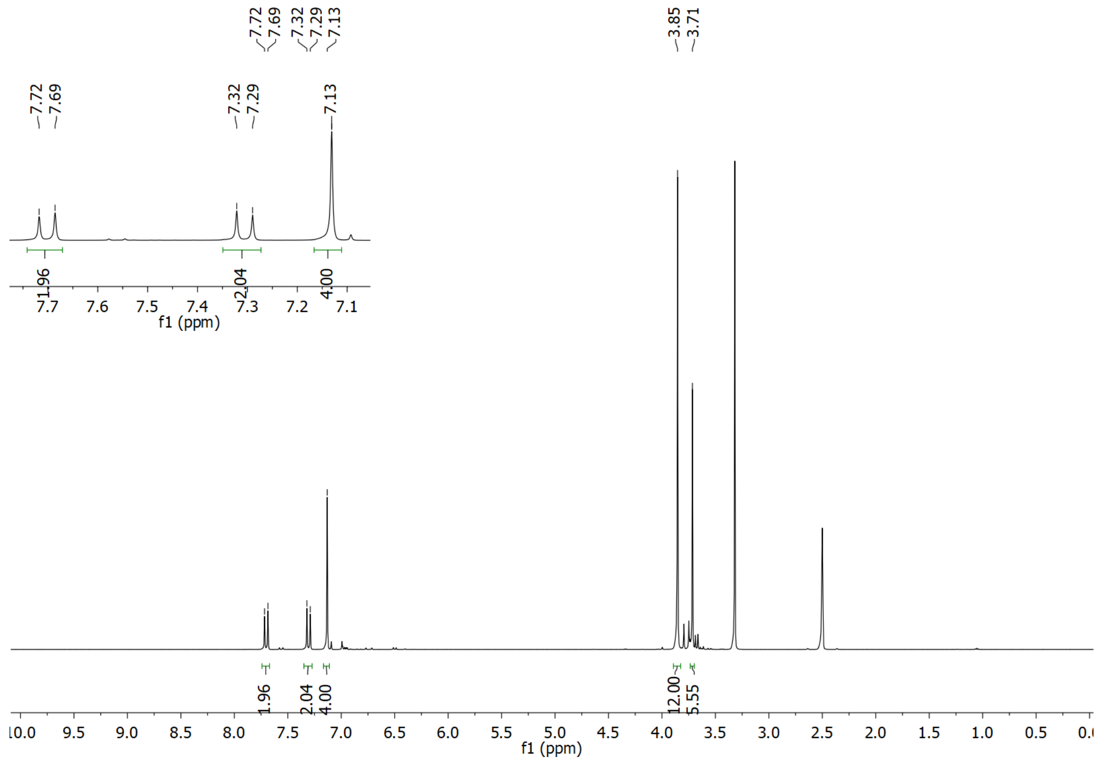

Figure S24. ^{1}H NMR spectrum of compound (1*E*,4*E*)-1,5-bis(3,4,5-trimethoxyphenyl)penta-1,4-dien-3-one **6e** in DMSO-d_6.

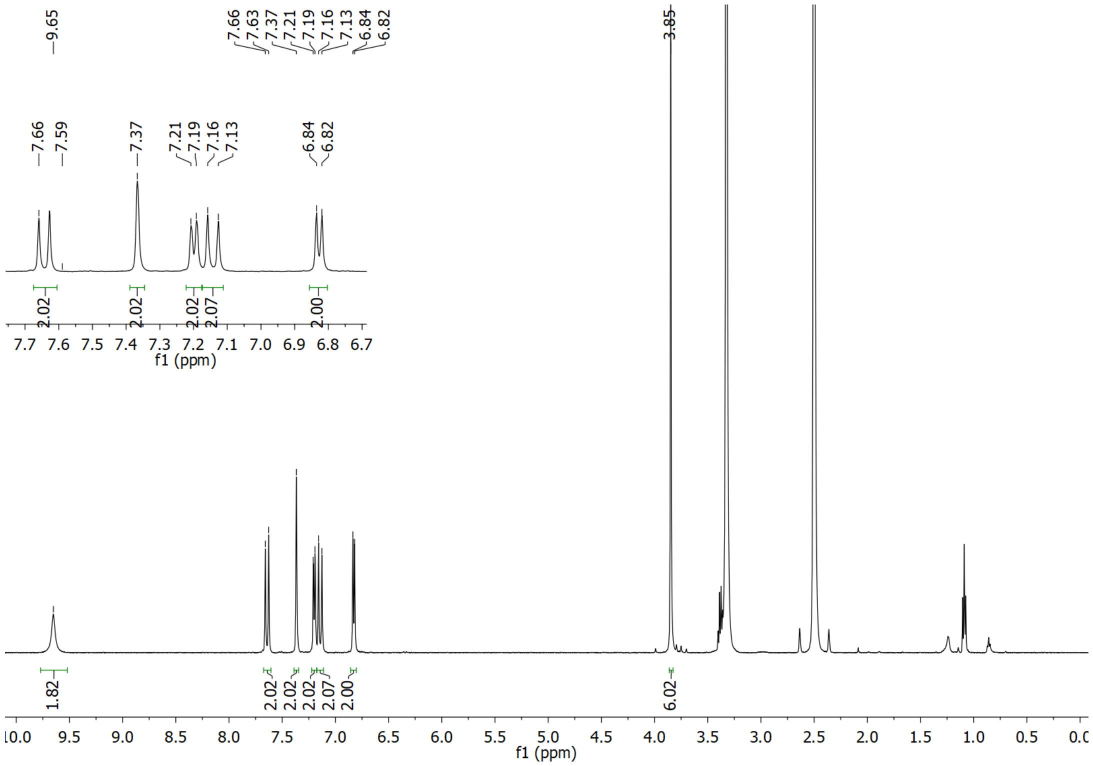

Figure S25. ^{1}H NMR spectrum of compound (1*E*,4*E*)-1,5-bis(4-hydroxy-3-methoxyphenyl)penta-1,4-dien-3-one **6f** in DMSO-d_6.

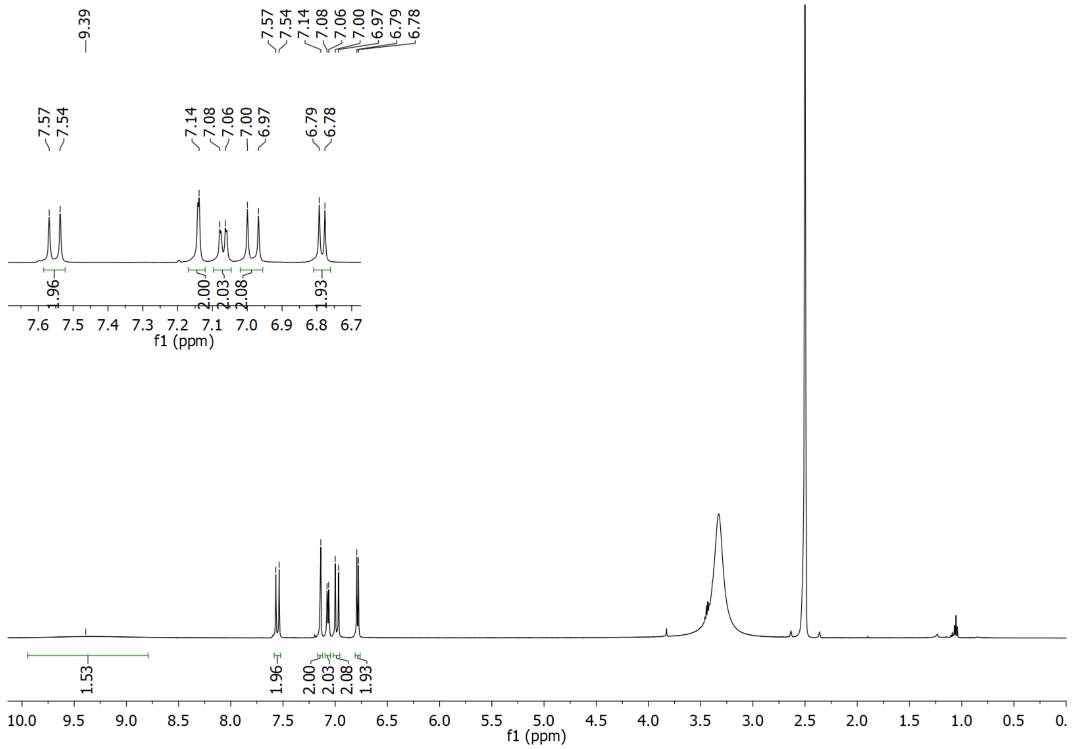

Figure S26. ^{1}H NMR spectrum of compound (1*E*,4*E*)-1,5-bis(3,4-dihydroxyphenyl)penta-1,4-dien-3-one **6g** in DMSO-d_6.

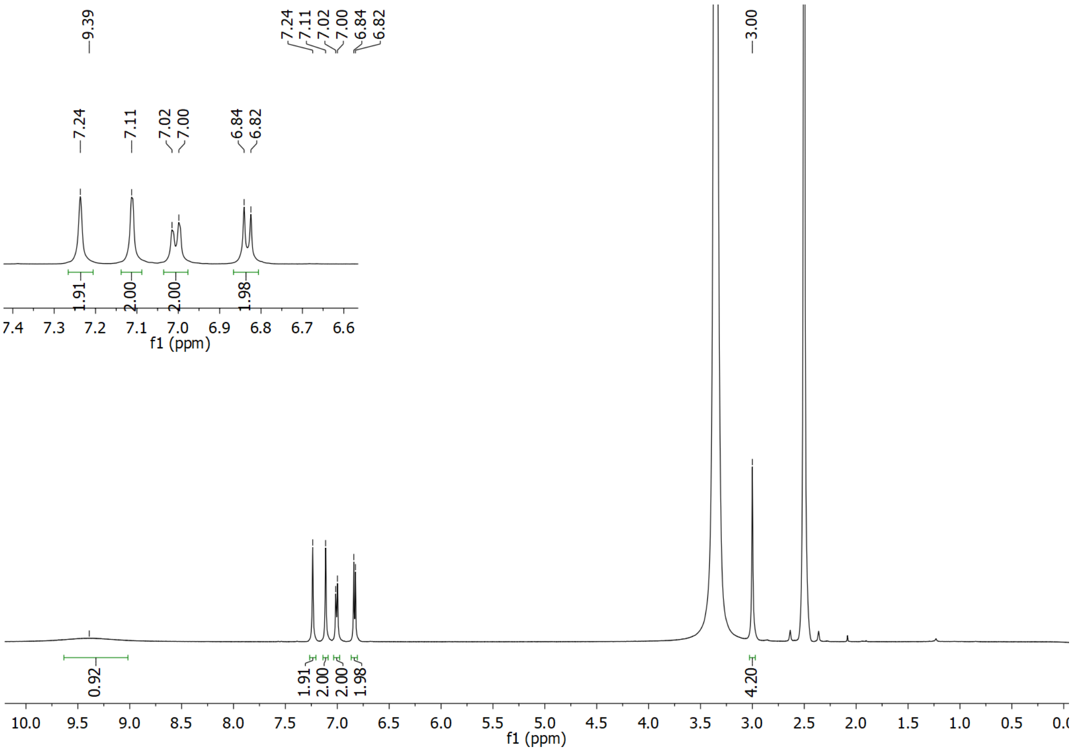

Figure S27. ^{1}H NMR spectrum of compound 2,5-bis((*E*)-3,4-dihydroxybenzylidene)cyclopentan-1-one **6h** in DMSO-d_6.

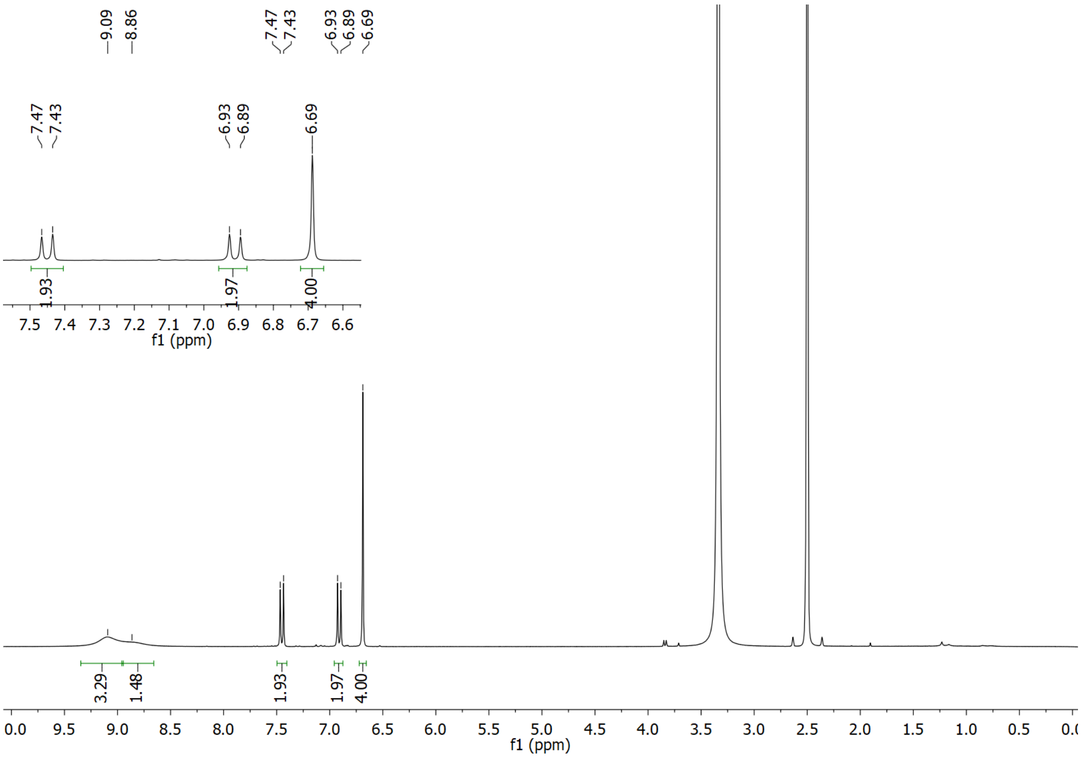

Figure S28. ^{1}H NMR spectrum of compound (1*E*,4*E*)-1,5-bis(3,4,5-trihydroxyphenyl)penta-1,4-dien-3-one **6i** in DMSO-d_6.

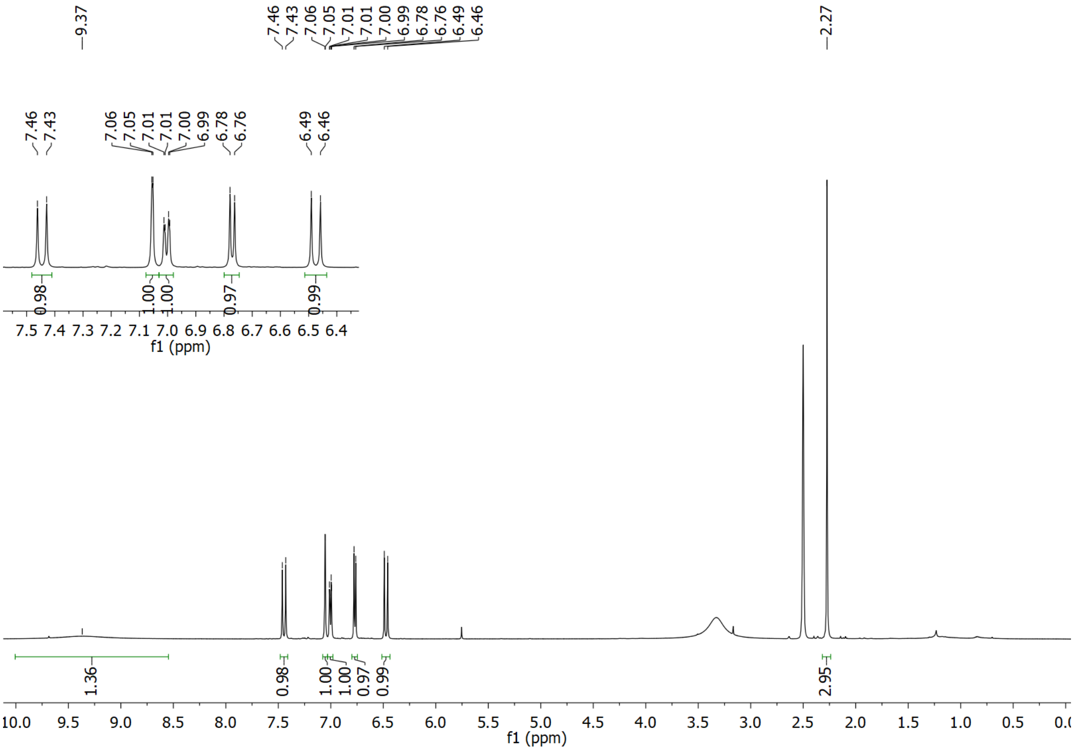

Figure S29. ^{1}H NMR spectrum of compound (*E*)-4-(3,4-dihydroxyphenyl)but-3-en-2-one **7a** in DMSO-d_6.

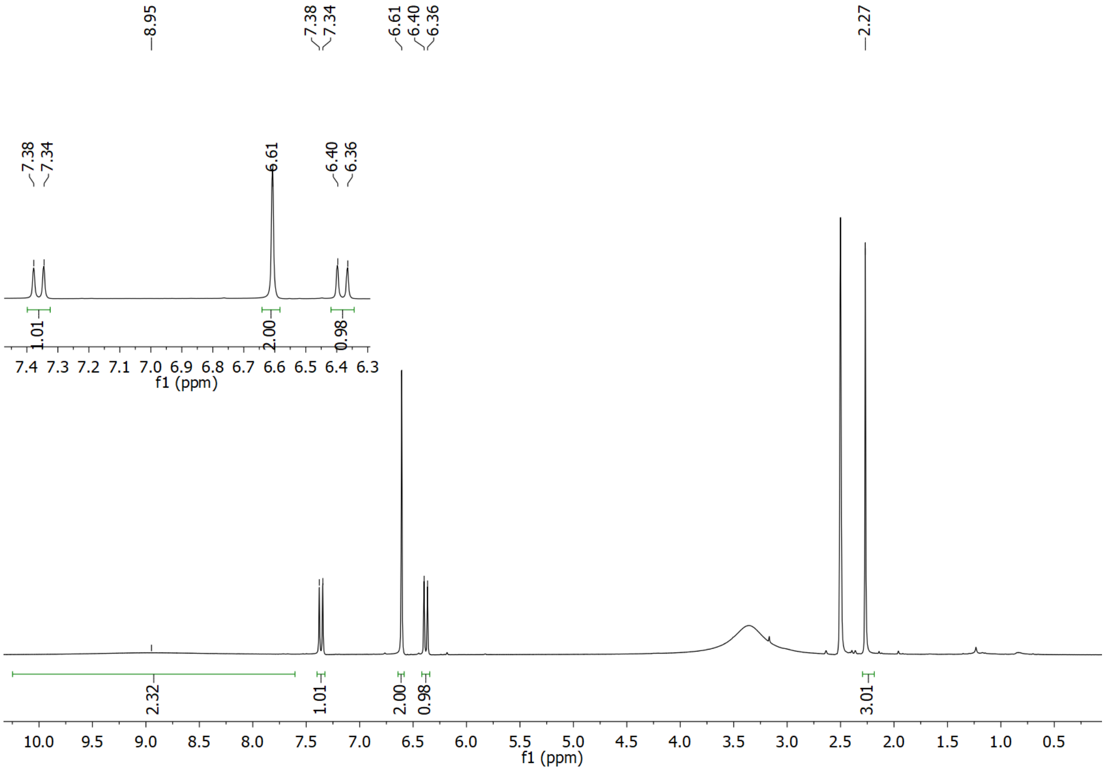

Figure S30. ^{1}H NMR spectrum of compound (*E*)-4-(3,4,5-trihydroxyphenyl)but-3-en-2-one **7b** in DMSO-d_6.
